# The *Aedes aegypti* siRNA pathway mediates broad-spectrum defense against human pathogenic viruses and modulates antibacterial and antifungal defenses

**DOI:** 10.1371/journal.pbio.3001668

**Published:** 2022-06-09

**Authors:** Yuemei Dong, Shengzhang Dong, Nahid Borhani Dizaji, Natalie Rutkowski, Tyler Pohlenz, Kevin Myles, George Dimopoulos

**Affiliations:** 1 W. Harry Feinstone Department of Molecular Microbiology and Immunology, Bloomberg School of Public Health, Johns Hopkins University, Baltimore, Maryland, United States of America; 2 Texas A & M University, Department of Entomology, TAMU College Station, Texas, United States of America; Radboud University Medical Center, NETHERLANDS

## Abstract

The mosquito’s innate immune system defends against a variety of pathogens, and the conserved siRNA pathway plays a central role in the control of viral infections. Here, we show that transgenic overexpression of Dicer2 (*Dcr2*) or *R2d2* resulted in an accumulation of 21-nucleotide viral sequences that was accompanied by a significant suppression of dengue virus (DENV), Zika virus (ZIKV), and chikungunya virus (CHIKV) replication, thus indicating the broad-spectrum antiviral response mediated by the siRNA pathway that can be applied for the development of novel arbovirus control strategies. Interestingly, overexpression of *Dcr2* or *R2d2* regulated the mRNA abundance of a variety of antimicrobial immune genes, pointing to additional functions of DCR2 and R2D2 as well as cross-talk between the siRNA pathway and other immune pathways. Accordingly, transgenic overexpression of *Dcr2* or *R2d2* resulted in a lesser proliferation of the midgut microbiota and increased resistance to bacterial and fungal infections.

## Abstract 2

This study shows that transgenic overexpression of siRNA pathway factors in mosquitoes mediates a broad-spectrum antiviral action against human pathogenic viruses such as dengue, Zika and Chikungunya virus, with implications for novel arbovirus control strategies; the siRNA pathway also regulates antimicrobial immune responses against bacterial and fungal infections.

## Introduction

Mosquito-transmitted arboviruses are increasingly causing reemergent epidemic disease worldwide, despite significant efforts to control their vectors over the past few decades. Dengue remains the major vector-borne viral disease and is responsible for over 390 million infections per year [1,2], and the Zika epidemics in 2015 and 2016 resulted in a tremendous public health burden. The lack of specific drugs and limited availability of a partially effective vaccine has accentuated the need for the development of novel control strategies for arbovirus transmission.

While dengue virus (DENV) and Zika virus (ZIKV) are flaviviruses, and chikungunya virus (CHIKV) is an alphavirus, all of these human pathogens are transmitted by *Aedes* mosquito species. The viral infection cycle in the mosquito starts in the midgut tissue with the acquisition of an infectious blood meal and ends in the salivary glands, from which transmission to another human host occurs through a second blood meal [3,4]. The mosquito infection cycle, or extrinsic incubation period, usually ranges from 3 to 14 days, depending on the mosquito and virus strains, as well as environmental factors [5–12]. To complete this journey, arboviruses utilize multiple mosquito-encoded host factors; at the same time, they are also hampered by mosquito-encoded restriction factors, which are often components of the insects’ innate immune system [5,12–14]. The mosquito’s classical innate immune signaling pathways, Toll and JAK-STAT, have been shown to exert antiviral activity, albeit not with equal potency against all arboviruses. For example, transgenic overexpression of the JAK-STAT positive regulators DOME and HOP in the fat body tissue results in specific suppression of infection with the DENV, but not with ZIKV or CHIKV [15]. Mosquitoes have a complex life cycle, and in nature, they are exposed to a variety of microbes, most of which are bacteria and fungi. The Toll and immune deficiency (Imd) pathways were originally identified as the insect’s main defense against these types of microbes [12,16,17].

While the small interfering RNA (siRNA) pathway in mosquitoes and fruit flies mediates an immune defense against RNA viruses through the key factors Dicer2 (DCR2), R2D2, and Argonaute2 (AGO2), among others, the breadth and specificity of its antiviral spectrum, as well as its tissue specificity, have not been addressed for mosquito-transmitted human pathogens [5,18–31]. Within mosquito cells, viral RNA is released through the acidification of endosomes into the cytoplasm where it is translated into a precursor polyprotein that is cleaved to form a replication complex producing negative RNA strands as templates for the replication of new viral genomes. The double-stranded viral RNA is recognized as a pathogen-associated molecular pattern (PAMP) by DCR2, followed by degradation of double-stranded RNA (dsRNA) into 21-bp siRNAs [14,23,24,32]. The double-stranded RNA binding protein (dsRBP) R2D2 then partners with DCR2 to load these siRNA products into an RNA-induced-silencing-complex (RISC), where one strand will be used as a guide to target AGO2 to complementary templates for degradation. Coimmunoprecipitation of *Aedes aegypti* DCR2 and R2D2 indicated that the partnership is also conserved in the mosquito [33].

Here, we have developed transgenic mosquitoes that overexpress either *Dcr2* or *R2d2* in the midgut tissue after a blood meal, in order to gain further insight into the functional roles of these 2 factors, and the siRNA pathway, in the interactions of *Ae*. *aegypti* with some of the most important pathogenic arboviruses DENV, ZIKV, and CHIKV, as well as other microbes and physiological systems. We show that *Dcr2* or *R2d2* overexpression in the midgut tissue results in an siRNA-mediated suppression of viral infection in this tissue and disseminated infection in the carcass and salivary glands. Furthermore, we demonstrate that these factors and the siRNA pathway also have diverse roles, including the regulation of antibacterial and antifungal defenses through a variety of antimicrobial peptides (AMPs) and other immune factors. The *Dcr2*- and *R2d2*- transgenic mosquitoes showed minimally compromised fitness when compared to wild-type controls. Our results indicate that the broad-spectrum antiviral activity of the siRNA pathway, taken together with the feasibility of its augmentation by *Dcr2* and *R2d2* overexpression, can be utilized for the development of novel arbovirus control strategies.

## Results

### Generation of blood meal–inducible *Dcr2-* and *R2d2*-expressing transgenic *Ae*. *aegypti*

**Fig 1:**
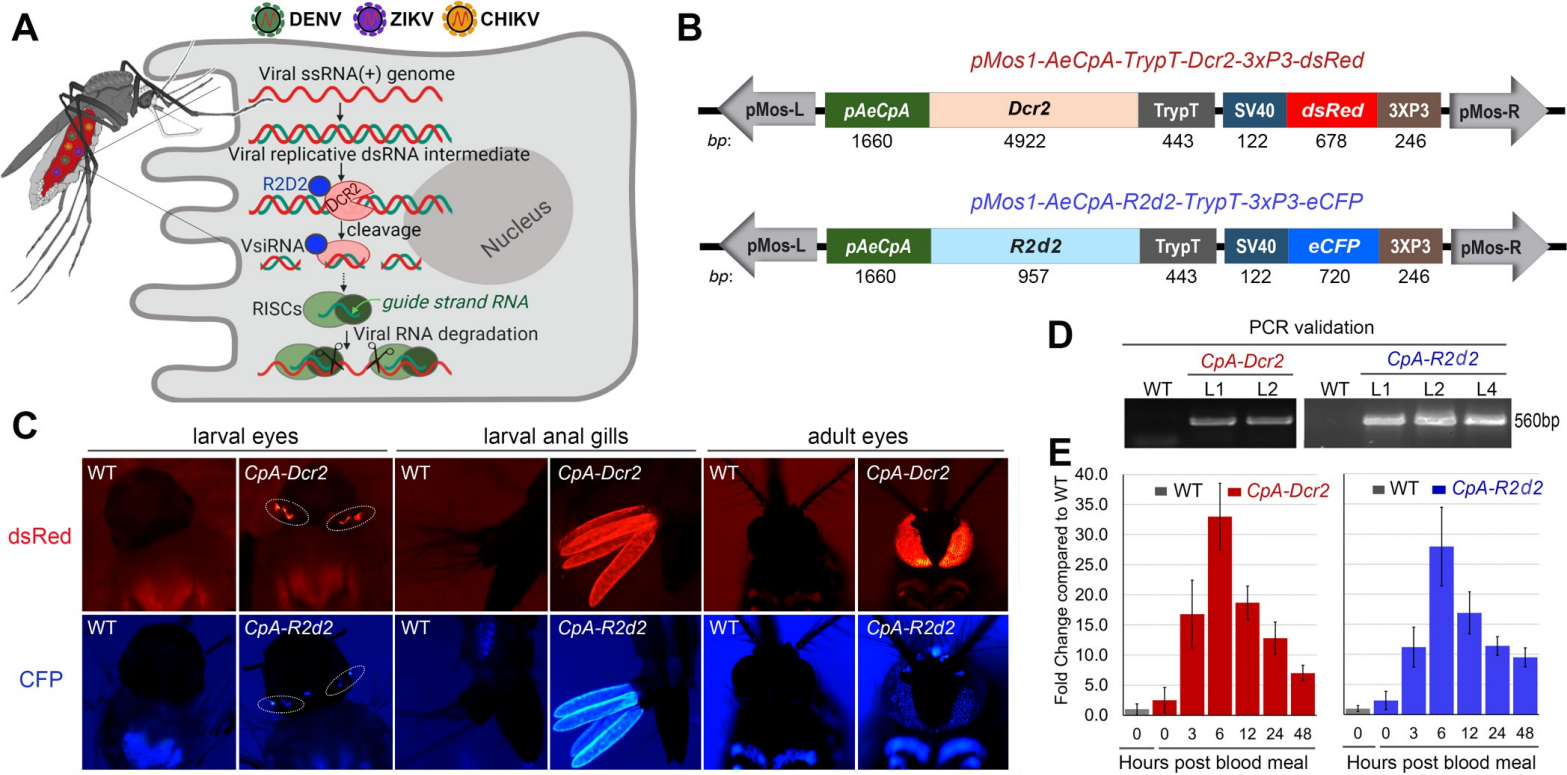
Generation of transgenic *Aedes* mosquitoes overexpressing *Dcr2* and *R2d2* under the control of the *carboxypeptidase A* promoter (*pAeCpA* or *CpA*). (**A**) Schematic presentation of the viruses in the blood bolus of the mosquitoes after an infectious blood meal and the mechanisms undergirding the antiviral siRNA pathway. In the cytoplasm of mosquito midgut epithelium cells, a viral replicative dsRNA intermediate is recognized by DCR2 in complex with R2D2, followed by cleavage of the viral dsRNA into 21-nt siRNAs by AGO2 in the RISC. The illustration was created with BioRender.com. (**B**) Schematic presentation of the 2 *Mos1-mariner* (*pMos1*) transformation plasmids used to generate the *CpA-Dcr2* and *CpA-R2d2* lines. *pMosL*, *pMosR*: *Mos1-mariner* left and right arms. 3xP3-eCFP and 3xP3-dsRed are used for eye-specific promoter-driven expression of either *eCFP* (blue) or *dsRed* (red) as eye markers for sorting the transgenic mosquitoes. *AeCpA* promoter: carboxypeptidase A promoter; *Dcr2* or *R2d2*: *Dcr2* or *R2D2* gene coding sequences; TrypT: trypsin terminator sequence. (**C**) Fluorescent images of larvae and adults of the *CpA-Dcr2* and *CpA-R2d2* transgenic mosquitoes. (**D**) Confirmation of transgenes through PCR using the primers listed in S1 Table. (**E**) Transcript abundance of transgenes (*Dcr2* in red and *R2d2* in blue) in the midgut of *CpA-Dcr2* and *CpA-R2d2* lines from before the blood meal (0 h) to 48 h PBM. Each bar represents the relative-fold change of the corresponding gene assayed through qRT-PCR in *CpA-Dcr2* or *CpA-R2D2*, compared between transgenic lines and WT mosquitoes. The *rps17* gene was used to normalize the cDNA template, and error bars indicate the S.E. of the mean. dsRNA, double-stranded RNA; PBM, post-blood meal; qRT-PCR, quantitative real-time PCR; siRNA, small interfering RNA; WT, wild type.

Studies have demonstrated the production of DENV-derived siRNAs and piwi-interacting RNAs in virally infected mosquitoes, suggesting that the RNA interference (RNAi) machinery is potent in controlling viral infection and replication (Fig 1A) [12,14]. In the siRNA immune pathway, both DCR2 and R2D2 are key players in assuring proper RNA recognition and activation of this pathway.

To investigate the roles of DCR2, R2D2, and the siRNA pathway in the mosquito’s midgut antiviral defense and other physiological systems, we generated transgenic mosquitoes overexpressing *Dcr2* (AY713296.1) and *R2d2* (KJ598053.1) under the control of the blood meal–inducible midgut-specific carboxypeptidase A (*AeCpA or CpA*, AF165923) promoter using primers listed in S1 Table [27,34]. Through a Gateway cloning system, we have generated 2 constructs for embryonic transformation: *pMos1-AeCpA-Dcr2-3xP3-dsRed* and *pMos1-AeCpA-R2d2-3xP3-eCFP* (Fig 1B and S1 Data). The fluorescent eye markers eCFP (for *R2d2*) and dsRed (for *Dcr2*) were used to enable the screening of positive larvae and adult mosquitoes (Fig 1C). Embryonic microinjection of the constructs in an injection mix with helper plasmid resulted in the generation of 2 *CpA-Dcr2*- and 7 *CpA-R2d2*-expressing transgenic *Ae*. *aegypti* lines after the screening of eye markers at the G_1_ generation (Fig 1C). The insertions of the corresponding transgene were confirmed by PCR on G_4_ mosquitoes (Fig 1D). At the G_4_ generation, all 9 heterozygous lines were infected with both DENV serotype 2 (DENV2) and ZIKV; the *CpA-Dcr2*-L2 (line #2) and *CpA-R2D2*-L2 (line #2) displayed the strongest resistance to both DENV2 and ZIKV, with lower infection intensity and prevalence (S1 and S2 Figs). Though the *CpA-Dcr2*-L1 (line #1) has shown slightly greater resistance to DENV2, as measured by infection prevalence (S1 Fig), the *CpA-Dcr2*-L2 (line #2) showed significant resistance to both ZIKV and DENV2 viruses and was therefore used for down-stream studies. The various heterozygous transgenic lines showed different degrees of resistance to both DENV2 and ZIKV (S1 and S2 Figs), suggesting positional effects on transgene activity. Inverse PCR, using the primers, listed in S1 Table was applied to amplify the flanking region of the pMos1 left arm region and the precise chromosomal locations of the transgenes on each transgenic line were confirmed through PCR product purification and sequencing. VectorBase (http://www.vectorbase.org) was searched for sequences corresponding to the junctions between transposon landing sites on *Ae*. *aegypti* genome and transposon arms using the BLASTn tool (S2 Table). The *CpA-Dcr2*-L2 and *CpA-R2d2*-L2 transgenic lines were selected for the generation of homozygous lines at G_8_ and are hereafter referred to as *CpA-Dcr2* (or abbreviated as *Dcr2* in the figures) and *CpA-R2d2* (or abbreviated as *R2d2*).

To validate the blood meal–inducible midgut-specific up-regulation of the *Dcr2* and *R2D2* transgenes, and to assess their temporal expression profiles, we applied quantitative real-time PCR (qRT-PCR) to measure their mRNA abundance at successive times (3, 6, 12, 24, and 48 h) after a blood meal. Both *Dcr2* and *R2d2* were significantly up-regulated at 6 to 12 h after a blood meal, with a 33- and 28-fold induction of *Dcr2* and *R2d2*, respectively (Fig 1E). The transgenes were still showing a 7- and 9.5-fold induction of *Dcr2* and *R2d2*, respectively, at 48-h post-blood meal (PBM). The continuous overexpression of these transgenes between 3 and 48 h PBM suggests that the siRNA pathway is likely augmented during the time window in which viruses establish an infection in midgut epithelial cells.

### Transgenic overexpression of *Dcr2* and *R2d2* in the midgut tissue inhibits DENV2, ZIKV, and CHIKV replication through viral RNA degradation

**Fig 2:**
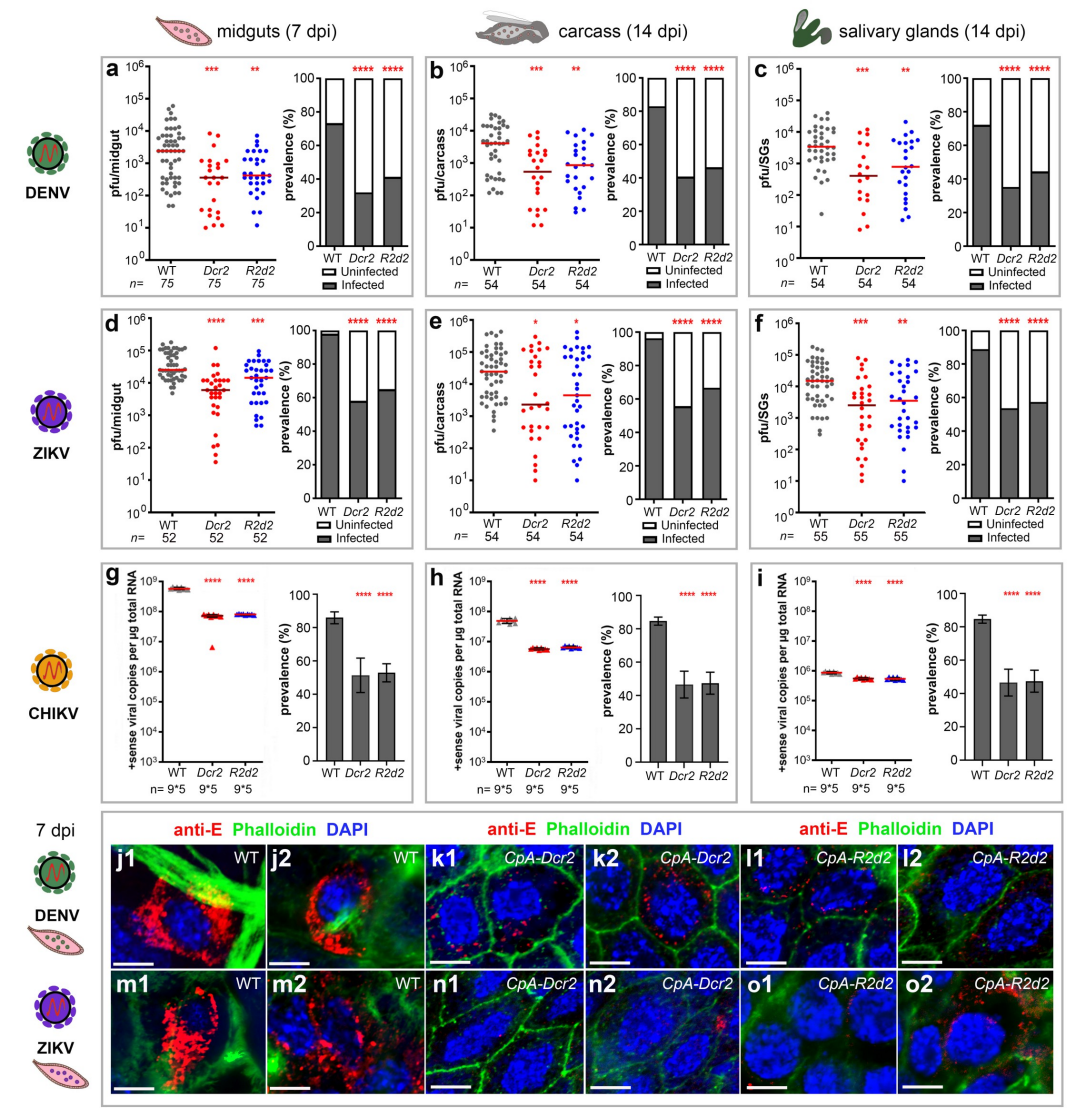
Antiviral effect of RNAi pathway activation on DENV2, ZIKV, and CHIKV infection in transgenic *CpA-Dcr2* (*Dcr2*) and *CpA-R2d2* (*R2d2*) *Ae*. *aegypti*. Overexpression of *Dcr2* and *R2d2* was induced in the transgenic mosquitoes when the infectious blood meal was given at time 0 h. (**a**–**i**) The viral infection titers and infection prevalence of DENV2, ZIKV (Cambodia), and CHIKV of the *CpA-Dcr2* and *CpA-R2d2* lines (DENV2: a–c; ZIKV: d–f; CHIKV: g–i) were measured after midgut infection at 7 days PIBM, and dissemination of the virus in the carcass and SGs at 14 days PIBM. Sibling WT *Ae*. *aegypti* Liverpool strain was used as a control in parallel in all experiments. Plaque assays were used to determine viral loads and infection prevalence for both DENV2 and ZIKV in individual mosquitoes, and qRT-PCR was used to determine CHIKV viral loads in a pool of 5 mosquitoes, along with CPE assay for the determination of infection and dissemination of virus. Horizontal red lines indicate the median of the viral loads. At least 3 replicates were pooled for the statistical analyses, using the Mann–Whitney test to compare median virus titers and Fisher’s exact test to compare infection prevalence. *P < 0.05, **P < 0.01, ***P < 0.001, ****P < 0.0001. (**j–o**) Confocal IFA with anti-envelope protein antibody (anti-E, red) shows that the WT midguts at 7 dpi were heavily infected with both DENV2 (j) and ZIKV (m) viruses. DAPI (blue) indicates the nuclei of the midgut epithelium cell, the phalloidin (green) indicates the actin cell skeleton. Scale bars, 5 μm. Data underlying this figure can be found in S2 Data. CHIKV, chikungunya virus; CPE, cytopathic effect; DENV2, DENV serotype 2; dpi, days post-infection; IFA, immunofluorescence microscopy assay; PIBM, post-infectious blood meal; qRT-PCR, quantitative real-time PCR; SG, salivary gland; ZIKV, Zika virus.

To obtain a similar genetic background of the transgenic mosquitoes with the wild type (WT) controls, we backcrossed the homozygous transgenic lines with the WT line for 4 generations and reobtained the homozygotes for down-stream analyses. The sibling WT mosquitoes from these screens were maintained side by side with the transgenic homozygotes thereafter and were used as WT controls. To investigate the impact of blood meal-induced overexpression of Dcr2 and R2D2 on arbovirus infection, we infected *CpA-Dcr2* and *CpA-R2d2* transgenic mosquitoes, along with sibling WT (referred to as WT onwards) as control, with DENV2, ZIKV, and CHIKV through artificial blood meals containing 10^6–7^, 10^8^, and 10^7–8^ PFU/ml virus particles, respectively (Fig 2), and then measured the infection intensity and prevalence in the midgut at 7 days post-infection (dpi) and the dissemination rate in the carcass and salivary glands at 14 dpi. The DENV2 infection intensities were significantly reduced in the midgut tissues of both *CpA-Dcr2* and *CpA-R2d2* mosquitoes when compared to the WT mosquitoes at 7 dpi (a significant 7-fold and 5-fold reduction in median titers measured through plaque assays for the *CpA-Dcr2* and *CpA-R2d2* lines, respectively) (Fig 2A, Mann–Whitney test, **P < 0.01, **P < 0.001). Infection prevalence also showed a significant 2.1- and 1.7-fold reduction in *CpA-Dcr2* and *CpA-R2d2* mosquitoes, respectively (Fig 2A, Fisher’s exact test, ****P < 0.0001). Both transgenic lines also displayed a significant reduction in median disseminated infection intensity in the carcass tissue (Fig 2B, Mann–Whitney test, **P < 0.01, ***P < 0.001) and salivary gland infection intensity (Fig 2C, Mann–Whitney test, **P < 0.01, ***P < 0.001) at 14 dpi. The DENV2 infection prevalence was also significantly reduced in the carcass tissue (Fig 2B, Fisher’s exact test, ****P < 0.0001) and salivary glands (Fig 2C, Fisher’s exact test, ****P < 0.0001) at 14 dpi.

We performed similar infection assays with ZIKV, another flavivirus family member. Both *CpA-Dcr2* and *CpA-R2d2* transgenic mosquito midguts displayed significantly lower ZIKV infection intensities than did WT control mosquitoes, with a 4.2- and 1.8-fold reduction in median viral titers for *CpA-Dcr2* and *CpA-R2d2*, respectively at 7 dpi (Fig 2D, Mann–Whitney test, ***P < 0.001, ****P < 0.0001). Infection prevalence was also significantly reduced, from 98.1% in WT to 57.7% (a 1.7-fold reduction) in *CpA-Dcr2* and 65.4% (a 1.5-fold reduction) in *CpA-R2d2* transgenic mosquitoes (Fig 2D, Fisher’s exact test, ****P < 0.0001). The infection intensities in carcasses at 14 dpi showed a statistically insignificant trend toward reduction (10.7- and 5.4-fold) when compared to WT mosquitoes for *CpA-Dcr2* and *CpA-R2d2*, respectively (Fig 2E, Mann–Whitney test, *P < 0.05). However, both transgenic mosquito lines showed a significantly lower ZIKV dissemination prevalence at 14 dpi, with a 42.3% and 30.8% reduction for *CpA-Dcr2* and *CpA-R2d2*, respectively (Fig 2E, Fisher’s exact test, ****P < 0.0001). At the salivary gland infection stage, *CpA-Dcr2* mosquitoes displayed a significant 6-fold reduction in ZIKV infection intensity (Fig 2F, Mann–Whitney test, ***P < 0.001) and 1.7-fold reduction in prevalence (Fig 2F, Fisher’s exact test, ****P < 0.0001), and *CpA-R2d2* mosquitoes showed a significant 5.8-fold reduction in ZIKV infection intensity (Fig 2F, Mann–Whitney test, **P < 0.01) and 1.5-fold reduction in prevalence (Fig 2F, Fisher’s exact test, ****P < 0.0001).

In an independent set of experiments, we also tested whether blood meal-induced overexpression of *Dcr2* and *R2D2* would exert an effect on infection with CHIKV, an arbovirus from the alphavirus genus. The CHIKV infection prevalence, measured by cytopathic effect (CPE) assay, showed a significant 40.2% and 38.5% reduction for *CpA-Dcr2* and *CpA-R2d2*, respectively (from 86% in WT to 51.4% in *CpA-Dcr2* and 52.9% in *CpA-R2d2*) (Fig 2G, Fisher’s exact test, ****P < 0.0001). The CHIKV infection intensity, measured by qRT-PCR of CHIKV positive-strand copies, showed a significant 7.8- and 7.0-fold reduction in the midgut tissue at 7 dpi for *CpA-Dcr2* and *CpA-R2d2* when compared to the WT controls, respectively (Fig 2G, Mann–Whitney test, ****P < 0.0001). As compared to the WT mosquitoes, both *CpA-Dcr2* and *CpA-R2d2* transgenic mosquitoes displayed a significant 8.7- and 7.0-fold, respectively, reduction in infection intensity in the carcass tissue at 14 dpi (Fig 2H, Mann–Whitney test, ****P < 0.0001). Disseminated infection prevalence decreased significantly from 84.7% in WT carcasses to 46.7% in *CpA-Dcr2* (1.8-fold reduction) and 47.5% in *CpA-R2d2* carcasses (1.78-fold reduction) (Fig 2H, Fisher’s exact test, ****P < 0.0001). The salivary gland CHIKV infection intensities of *CpA-Dcr2* and *CpA-R2d2* transgenic mosquitoes showed a significant reduction of 1.6- and 1.7-fold, respectively, when compared to the control WT mosquitoes (Fig 2I, Mann–Whitney test, ****P < 0.0001). The disseminated infection prevalence in the salivary glands decreased significantly in both lines (Fig 2I, Fisher’s exact test, ****P < 0.0001). These data show that blood meal-induced *Dcr2* and *R2d2* expression in the midgut tissue also suppresses infection with CHIKV, likely through augmenting the activity of the siRNA pathway, and suggests a broad-spectrum protective role for the siRNA pathway in anti-viral immunity.

To visualize the viral infection patterns in the midgut tissue, we used confocal immunofluorescence microscopy (IFA) to assay the viral infection of DENV2 or ZIKV at 7 dpi in the transgenic *CpA-Dcr2* and *CpA-R2d2* mosquito midguts along with WT midguts as controls. The WT mosquito midguts were heavily infected with both DENV2 (Fig 2J–2L) and ZIKV (Fig 2M–2O), whereas the transgenic midguts had much less staining of viruses; these results agreed with those obtained by plaque assay and IFA staining showed a similar pattern as observed in previous studies [7,25], demonstrating the viral infections were significantly inhibited in both *CpA-Dcr2* and *CpA-R2d2* transgenic mosquito midguts (Fig 2).

**Fig 3:**
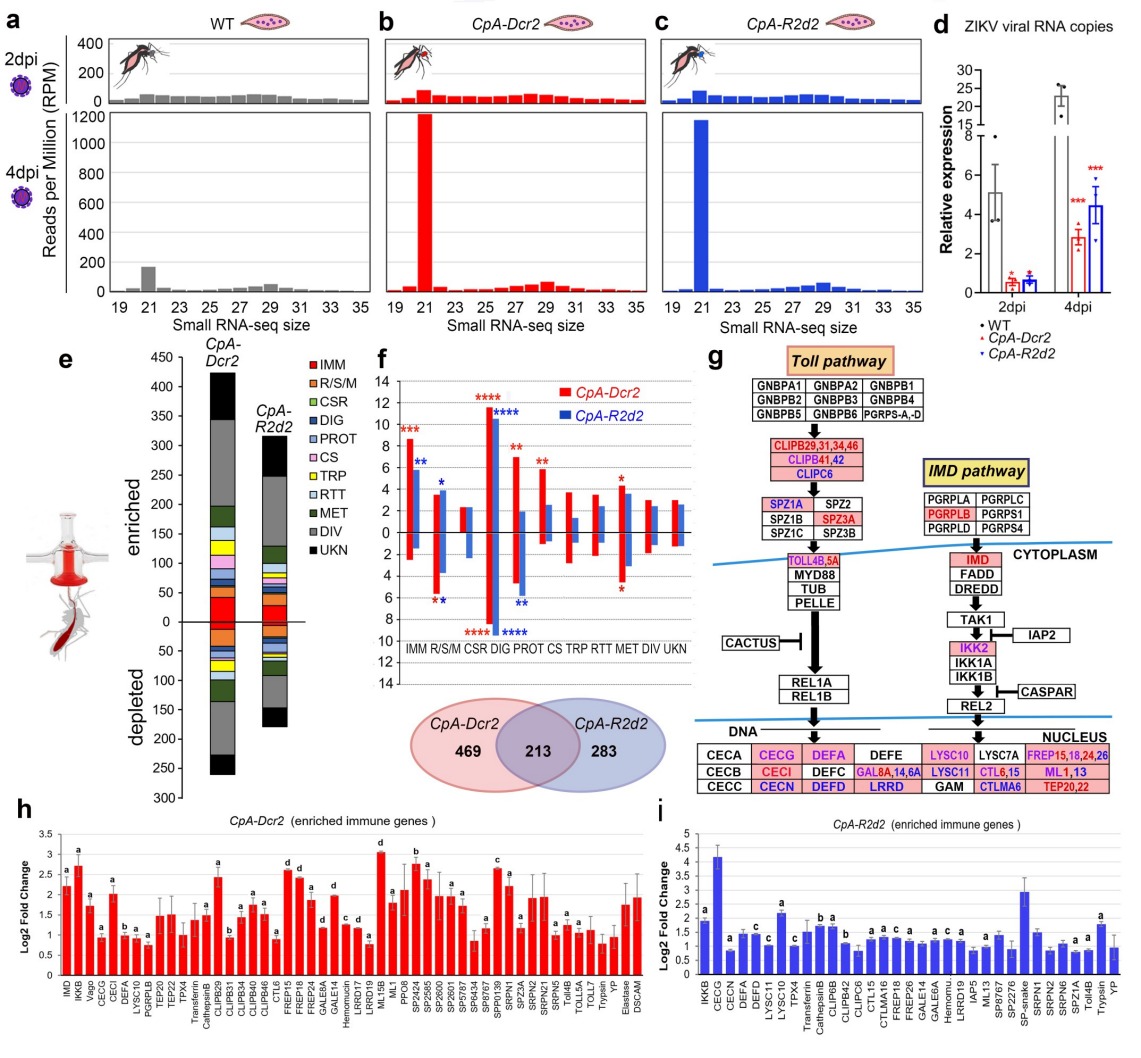
Production of ZIKV-derived siRNAs in infected WT and transgenic mosquitoes, and RNA-seq transcriptomic analysis of *CpA-Dcr2* and *CpA-R2d2* transgenic mosquitoes as compared to WT at 24 h PBM. (**a–c**) WT and transgenic (*CpA-Dcr2* and *CpA-R2d2*) mosquitoes were given a ZIKV-infected blood meal, and midgut samples were collected at 2 and 4 dpi for small RNA sequencing. Size distribution and 5′ base frequency of ZIKV-derived small RNA sequences from 3 mosquito groups at 2 time points. (**d**) Relative expression of total ZIKV RNA, as a measure of viral load, in the control (WT), *CpA-Dcr2*, and *CpA-R2d2* midguts at 2 or 4 dpi. The *AeRps7* gene was used as internal control with at least 3 biological replicates, the bar indicates the mean value +/− SEM. (**e**) The number of DEGs within each FG between the midguts of transgenic and WT. (**f**) For each FG, the percentage of enriched or depleted transcripts in the midguts of transgenic lines as compared to WT. The total number of significant DEGs in both *CpA-Dcr2* and *CpA-R2d2* mosquitoes is shown in the Venn diagram. (**g**) Pathway analysis of both Toll and IMD pathways indicates immune genes in these pathways are strongly up-regulated. The boxes colored in pink represent genes up-regulated in either or both pathways, letters in red or blue or purple indicate genes up-regulated in *CpA-Dcr2* or *CpA-R2d2*, respectively, or in both transgenic lines. (**h** and **i)** Log_2_-fold change in up-regulation of immune genes in either *CpA-Dcr2* (h) or *CpA-R2d2* (i) transgenic mosquitoes. Three biological replicates are given for each gene. The bar indicates the mean value +/− SEM. a, *P <* 0.05; b, *P <* 0.01; c, *P <* 0.001; d, *P <* 0.0001. Detailed information of these up-regulated genes is listed in S3 Table. The illustration was created with BioRender.com. Data underlying this figure can be found in S2 Data. CS, cytoskeletal and structural; CSR, chemosensory reception; DEG, differentially expressed gene; DIV, diverse functions; DIG, digestive; dpi, days post-infection; FG, functional group; IMM, immunity; MET, metabolism; PBM, post-blood meal; PROT, proteolysis; RSM, redox/stress/mitochondrion; RTT, replication/transcription/ translation; TRP, transport; UKN, unknown functions; WT, wild type; ZIKV, Zika virus.

Next, we asked whether the suppression of viral infection in the midgut tissue upon transgenic overexpression of *Dcr2* and *R2d2* was mediated by the siRNA pathway. To answer this, we performed deep sequencing of small RNAs from both transgenic mosquitoes and WT mosquito midguts 2 and 4 days after ingestion of a ZIKV-infected blood meal (Fig 3A–3C), and the relative expression of total viral RNA loads was measured through qRT-PCR, indicating significant suppression of the viral loads in the midguts of both *CpA-Dcr2* and *CpA-R2d2* transgenic lines at 2 or 4 dpi (Fig 3D). As controls, transgenic and WT mosquitoes were given a noninfectious blood meal, and WT mosquitoes were given an infectious blood meal with the same ZIKV titer given to the transgenic mosquitoes (Fig 3A). The data from the naïve blood meal controls showed no reads derived from the virus and were therefore excluded from the figure. The numbers of the virus-derived small RNAs (21-nt) in the infected mosquito midguts increased with time (Fig 3A–3C). We observed a significant enrichment of ZIKV-derived 21-nt small RNAs with a symmetrical distribution between the sense and antisense strands. Increased accumulation of 21-nt ZIKV siRNAs in the midguts of the *CpA-Dcr2* and *CpA-R2d2* mosquitoes at 4 dpi (Fig 3B and 3C) indicated that overexpression of *Dcr2* and *R2d2* in the midgut augmented the activity of the siRNA pathway restricting replication of the virus in this tissue.

### The siRNA pathway modulates antibacterial and antifungal defenses through the regulation of antimicrobial innate immune pathways and factors

While RNAi is known to encompass processes utilized by eukaryotes to modulate gene expression, maintain genome integrity, and defend against viruses, potential functions of the *Aedes* siRNA pathway beyond antiviral defense have not been adequately explored, particularly, the possibility of interactions with other components of the innate immune system. To obtain a broader understanding of which mosquito physiological systems are affected by the overexpression of *Dcr2* and *R2d2* and augmentation of the siRNA pathway, we used RNA-seq to compare the midgut transcriptomes of *CpA-Dcr2* and *CpA-R2d2* to that of WT mosquitoes at 24 h post-naïve blood meal. As expected, both *Dcr2* and *R2d2* transcripts were significantly enriched in the midguts of transgenic lines, along with hundreds of other differentially expressed genes (DEGs) belonging to diverse functional groups, indicating that DCR2, R2D2, and the siRNA pathway are influencing a variety of physiological functions.

In *CpA-Dcr2* mosquitoes, 423 and 259 transcripts were enriched or depleted, respectively, in the midguts when compared to the WT control (Fig 3E). In *CpA-R2d2* transgenic mosquitoes, 316 and 179 transcripts were enriched or depleted, respectively, when compared to the control (Fig 3E). As expected, a significant number (213) of transcripts were similarly regulated in both *CpA-Dcr2* and *CpA-R2d2* mosquitoes when compared to WT control mosquitoes (Fig 3F), with 130 of them being enriched and 83 being depleted when compared to the control (Fig 3F). These 213 commonly regulated genes most likely reflect an influence by the siRNA pathway rather than overexpression of *Dcr2* or *R2d2* independently. Gene ontology (GO) analysis using the GOstats package in R revealed an overrepresentation of cell cycle-related genes in this group. Interestingly, as many as 469 and 283 genes were specifically regulated in either *CpA-Dcr2* or *CpA-R2d2* mosquitoes, respectively, indicating that these siRNA pathway factors also play other unique roles in mosquito biology that may not be directly related to the function of the siRNA pathway.

We found a significant representation of innate immune genes (IMM functional group) and cytoskeletal and structural genes (CS functional group) among the differentially expressed (DE) transcripts, especially when the diverse (DIV) and unknown (UKN) functional groups were excluded from the analysis (Fig 3F). Given the incomplete GO annotation of *Ae*. *aegypti* transcripts in Vectorbase, we also performed a manual annotation using the Phyper package in R [35]. Among the total 62 DE immune-related genes, 77% were up-regulated and 23% were down-regulated, indicating cross-talk among DCR2 and R2D2 (key players of the siRNA pathway) and other pathways and factors of the innate immune system, including those that play roles in antibacterial and antifungal defense.

The 213 genes that were similarly regulated in both *CpA-Dcr2* and *CpA-R2d2* as compared to WT mosquitoes included 50 immune-related genes that encode AMPs, pattern recognition receptors (PRRs), immune pathway genes, clip-domain serine proteases, lysozyme, an MD-2 like protein, and other proteins. When we subjected the up-regulated immune-related genes to KEGG pathway analysis, we found several Toll and IMD pathway-related genes in both transgenic lines (Fig 3G), with 36 enriched transcripts in the *CpA-Dcr2* line, 21 enriched transcripts in the *CpA-R2d2* line, and 14 transcripts being enriched in both lines (Fig 3H and 3I and S3 Table). Overexpression of transgenes resulted in a more profound up-regulation of immunity-related genes in the *CpA-Dcr2* line than in the *CpA-R2d2* line (Fig 3G–3I), indicating a likely broader role for DCR2 in innate immunity. The regulation of numerous Toll and IMD pathway-related genes, especially genes encoding AMPs, suggests a possible involvement of DCR2, R2D2, and the siRNA pathway in regulating defenses against microbes such as bacteria and fungi.

**Fig 4:**
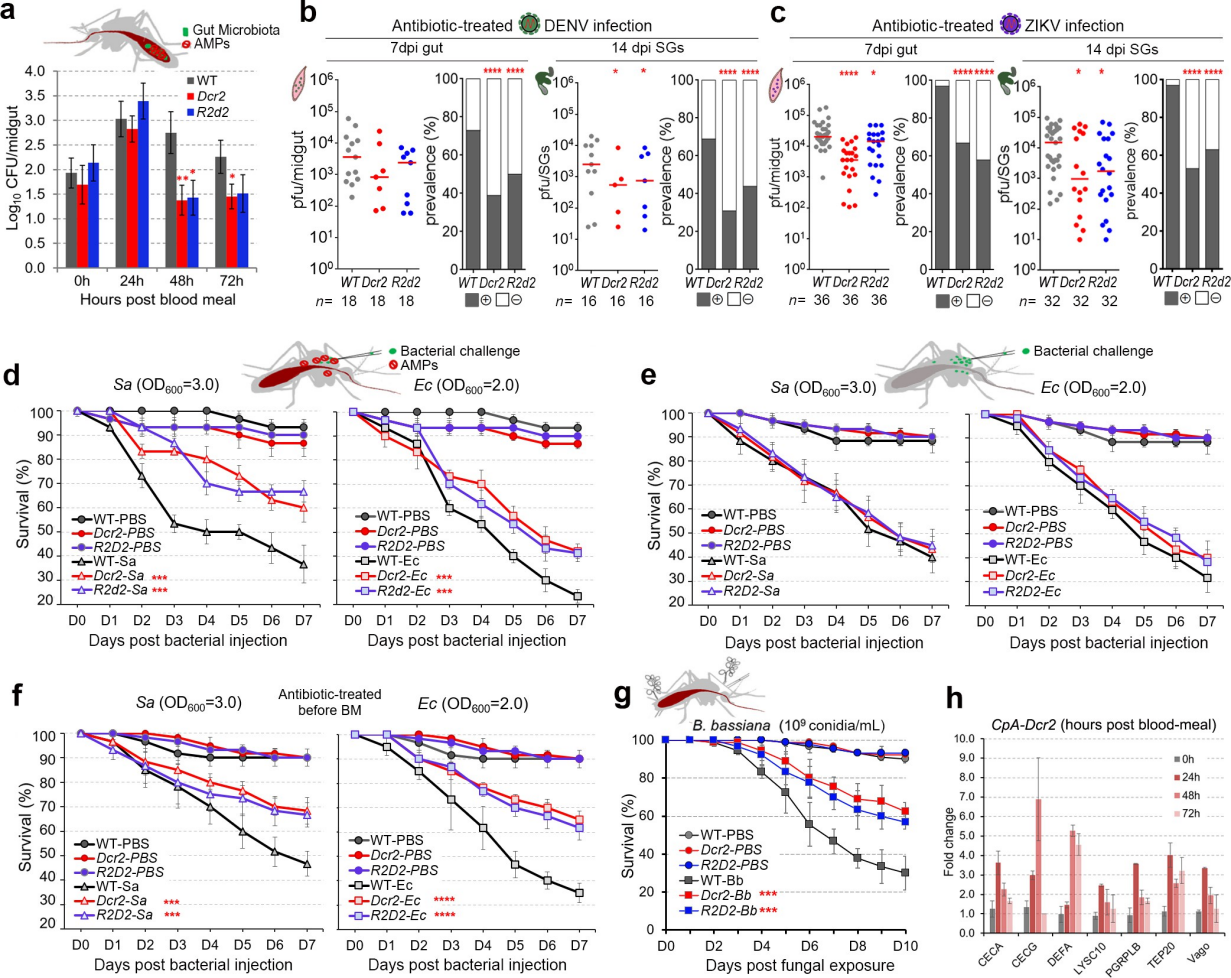
Antimicrobial resistance of siRNA pathway (*Dcr2* and *R2d2*) overexpressing transgenic mosquitoes (*Dcr2*: *CpA-Dcr2*, *R2d2*: *CpA-R2d2*). The up-regulation of *Dcr2* and *R2d2* in the transgenic mosquitoes activates the expression of several AMPs and then modulates the *Aedes* mosquitoes’ susceptibility to bacterial and fungal infection. (**a**) The total bacterial loads of the midgut microbiota of female transgenic and WT control mosquitoes at 24, 48, and 72 h PBM (mean ± SEM). A Student *t* test was used to determine significance. At least 3 biologicals were included with 10 mosquitoes in each replicate. (**b** and **c**) DENV2 (b) and ZIKV (c) viral infection intensities and infection prevalence in the aseptic (antibiotic-treated) transgenic and WT mosquitoes at 7 dpi (midguts) and 14 dpi (SGs) showed a similar level of reduction to that in the septic (non-antibiotic-treated) mosquitoes (as shown in Fig 2). At least 3 replicates are included, with each dot representing the viral load and the horizontal line (red) indicating the median value. The Mann–Whitney test was used to assess infection intensity, and the Fisher’s exact test was used to determine the significance of infection prevalence (* *P <* 0.05, **** *P <* 0.0001). (**d–f**) Survival rate of female transgenic mosquitoes after challenge with either gram-positive (*S*. *aureus*: 210,000 CFU) or gram-negative (*E*. *coli*: 140,000 CFU) bacteria at 7 dpi post a blood meal (d), or without a blood meal (e), or treated with antibiotics for 4 days followed by a blood meal 24 h before bacterial injection (f). (**g**) Survival rates of female WT, transgenic *CpA-Dcr2* and *CpA-R2d2* mosquitoes after *B*. *bassiana* infection. Prior to assays in (a, d, f, and g), all groups of the female mosquitoes were given a naïve blood meal to up-regulate the expression of the transgenes. The significance of the survival rates was determined by Kaplan–Meier survival analysis from 3 biological replicates with at least approximately 20 to 30 mosquitoes in each replicate (*** *P <* 0.001, **** *P* < 0.0001). (**h**) qRT-PCR expression profiling of a panel of antimicrobial and immune genes (S3 Table) in the midguts of female *CpA-Dcr2* transgenic mosquitoes at 0 h (before a blood meal), 24, 48, and 72 h PBM. The WT midguts were used as the control, and the *AeRps17* gene was used as an internal control for normalization. Data underlying this figure can be found in S2 Data. AMP, antimicrobial peptide; CFU, colony-forming unit; DENV2, DENV serotype 2; dpi, days post-infection; PBM, post-blood meal; qRT-PCR, quantitative real-time PCR; SG, salivary gland; siRNA, small interfering RNA; WT, wild type; ZIKV, Zika virus.

The mosquito intestine contains a diverse collection of bacteria, represented by the midgut microbiota, which is continuously controlled by the innate immune system to avoid over-proliferation, especially after a blood meal [15,36,37]. Interestingly, the midgut microbiota of both *CpA-Dcr2* and *CpA-R2d2* mosquitoes was suppressed (as determined by colony-forming unit [CFU] assays) after blood feeding, indicating that the up-regulation of these 2 siRNA pathway factors augments the antibacterial immunity in the midgut tissue, likely through the expression of AMP genes (Fig 4A). Suppression of the midgut microbiota was only observed at 48 and 72 h PBM, and not at 24 h, suggesting an indirect regulation of antimicrobial defenses by DCR2 and R2D2, and excluding the possibility that these 2 siRNA factors exert direct antimicrobial control (Fig 4A). As our CFU assays only detected culturable bacteria, we also measured the microbial load of the midguts by qRT-PCR analysis of 16s rRNA, which further confirmed suppression of the midgut microbiota in *CpA-Dcr2* and *CpA-R2d2* mosquitoes at 48 and 72 h PBM (S3 Fig).

We and others have previously shown that the mosquito midgut microbiota primes the immune system and stimulates basal immune activity through immune pathways that also control arboviral infection in mosquitoes [15,37,38]. To investigate whether the transgenic up-regulation of *Dcr2* or *R2d2* that mediated suppression of the microbiota also influences antiviral activity, we performed identical DENV2 and ZIKV infection assays with the *CpA-Dcr2*, *CpA-R2d2*, and WT mosquitoes under near germ-free conditions (or here called aseptic conditions, achieved through antibiotic treatment), then assayed viral titers in the midguts at 7 dpi and salivary glands at 14 dpi. The DENV2 and ZIKV infections were suppressed in the midguts and salivary glands of aseptic mosquitoes to levels that were similar to those of septic (non-antibiotic-treated) mosquitoes (arboviral infections in Fig 4B and 4C) were compared with those in Fig 2, Mann–Whitney test for infection intensity, Fisher’s exact test for infection prevalence, *P < 0.05, ***P < 0.001, ****P < 0.0001. These data show that the antiviral activities derived from the overexpression of *Dcr2* or *R2d2* are largely unaffected by DCR2- and R2D2-mediated suppression of the microbiota, suggesting that the siRNA-mediated antiviral defenses are independent of and predominant than microbiota-mediated antiviral activities.

We also investigated whether transgenic expression of *Dcr2* or *R2d2* in the midgut tissue influences mosquito survival after systemic bacterial infection. Indeed, a greater percentage of both *CpA-Dcr2* and *CpA-R2d2* female mosquitoes survived after thoracic injection of live gram-negative *Escherichia coli* (*Ec*) and gram-positive *Staphylococcus aureus* (*Sa*) than did WT female mosquitoes when the transgenes were induced upon a blood meal (Fig 4D, ***P < 0.*001*). The control groups (both PBS-injected WT and PBS-injected transgenic mosquitoes) did not differ from each other in survival rate. To ensure that the antibacterial activities are actually derived from the activation of the siRNA pathway, we have also studied the survival rates of these transgenic mosquitoes upon systemic bacterial challenge without induction of the transgenes (*Dcr2*, *R2d2*). The mortality rate between transgenic and control mosquitoes did not differ in the absence of blood meal–inducible transgene activation (Fig 4E). To exclude a possible indirect effect mediated by microbiota changes upon activation of the transgenes (Fig 4A), the mosquitoes were treated with antibiotics to be rendered near-aseptic prior to the blood meal. Both transgenic mosquito lines showed significantly better survival than the WT control upon either *Ec* or *Sa* challenges (Fig 4F, ***P < 0.*001*, ****P < 0.*0001*), similarly to what we had observed with non-antibiotic-treated septic mosquitoes (Fig 4D). Together, the results show that the antibacterial effects of the transgenic mosquitoes were indeed attributed to transgenes expression. Hence, the resistance to systemic infection with *Ec* or *Sa* is independent of changes in the microbiota. We have also orally challenged both control and transgenic mosquitoes with *Ec* or *Sa*, with or without blood meal activation of the transgenes, and the survival remained unaffected in either condition, likely due to the different infection routes of these tested bacteria (S4 Fig).

The Toll pathway also plays an important role in antifungal defense, and transgenic *Dcr2*- and *R2d2*-overexpression also resulted in the regulation of several Toll pathway-related immune genes (Fig 3G–3I). To assess whether *CpA-Dcr2* and *CpA-R2d2* mosquitoes possess an enhanced antifungal activity, we challenged these mosquitoes and WT mosquitoes with the entomopathogenic *Beauveria bassiana* (*B*. *bassiana*) 24 h after a naïve blood meal (to activate transgenes) administered through exposure to a conidial solution (10^9^ conidial/ml), according to previously established protocols [38]. Survival of both *CpA-Dcr2* and *CpA-R2d2* mosquitoes was significantly greater than that of WT control mosquitoes after fungal challenge, with a median LT_50_ > 10 days, as compared to 6.5 days for WT control mosquitoes (Fig 4G, Kaplan–Meier survival analysis, ***P < 0.001). These results agree with our previous observations of a relationship between antifungal and anti-DENV2 defenses, which is likely mediated by the Toll pathway [38].

To better understand the factors and mechanisms contributing to the microbiota changes and antibacterial or antifungal resistance upon transgene activation, we assayed the expression of a panel of immune genes, including antimicrobial factors, (CECA, CECG, DEFA, LYSC10, PGRP-LB, TEP20, Vago) in both midguts and carcass of WT control, *CpA-Dcr2*, and *CpA-R2d2* transgenic mosquitoes at 24, 48, and 72 h post blood meal (detailed information of these genes are available in S3 Table). Several of the immune genes were significantly up-regulated in both midgut and carcass tissues at 24 and 48 h post blood meal (Figs 4H and S5) in the transgenic mosquitoes, showing a positive correlation between the expression of immune genes and antimicrobial resistance.

### Transgenic *Dcr2* and *R2d2* expression negatively affects mosquito fecundity but enhances adult longevity

**Fig 5:**
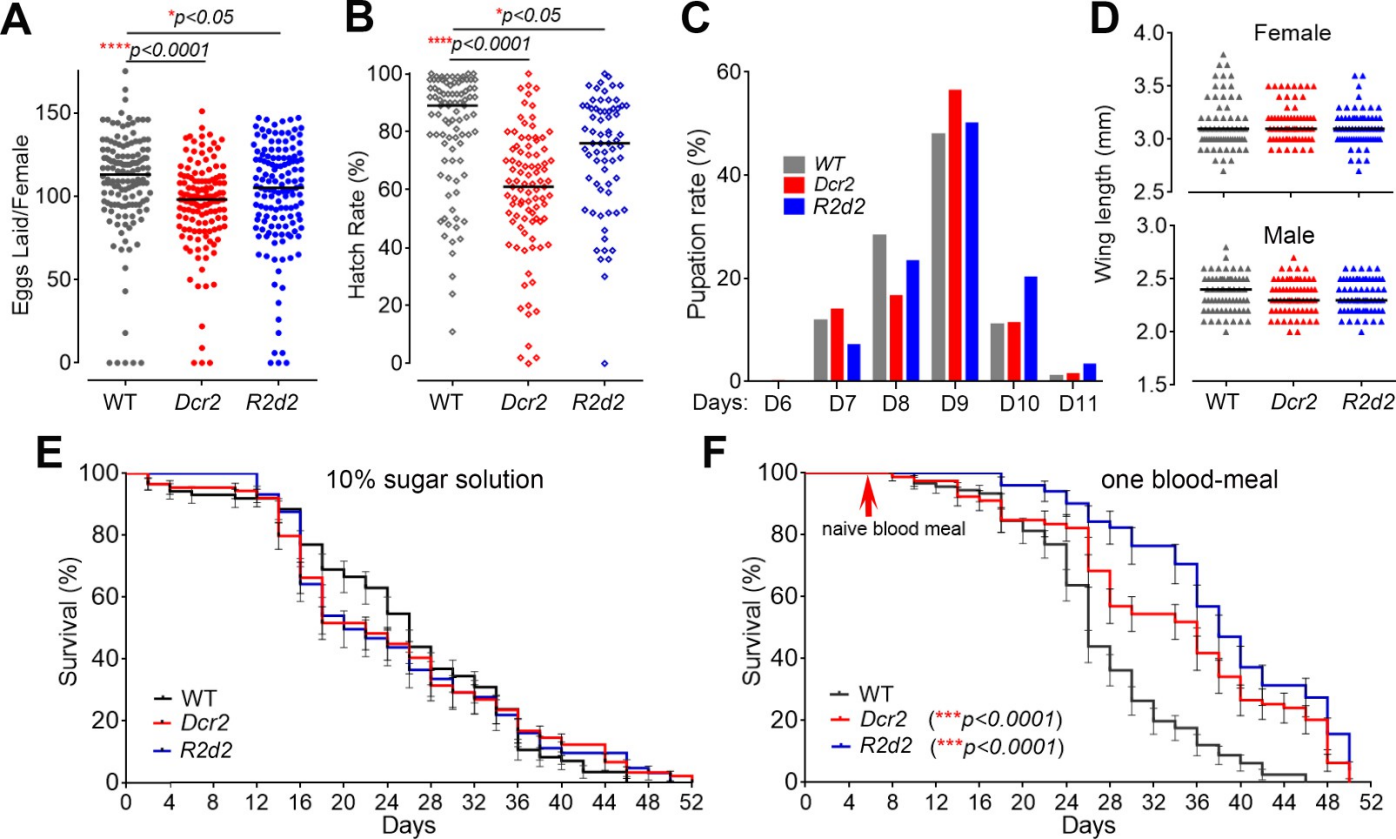
Fitness cost of the overexpression of *Dcr2* and *R2D2* in the midgut under the *carboxypeptidase* gene promoter (*AeCpA*) after a blood meal. *CpA-Dcr2* and *CpA-R2d2* transgenic mosquitoes are abbreviated as *Dcr2* and *R2d2*, respectively. (**A**) Fecundity of WT and transgenic *Ae*. *aegypti*, as represented by the number of eggs produced by each female mosquito. (**B**) Hatching rates of the transgenic mosquitoes’ eggs as compared to WT control Liverpool mosquitoes. Statistical analyses were performed using the Mann–Whitney test with GraphPad Prism 8 software with 3 biological replicates and 50 female mosquitoes in each replicate in (a, b). (**C**) Pupation rates of transgenic *CpA-Dcr2* and *CpA-R2d2 Ae*. *aegypti* mosquitoes. Two hundred larvae in each biological replicate, at least 3 biological replicates were included. (**D**) Wing lengths as a measure of the adult transgenic mosquito body size showed no differences between female or male transgenic *CpA-Dcr2* or *CpA-R2d2* mosquitoes and WT adult mosquitoes. Twenty mosquitoes for each biological replicate and 3 biological replicates were included. (**E** and **F**) Lifespan of female mosquitoes maintained on 10% sucrose solution and those of female mosquitoes provided 1 blood meal to induce the expression of the transgenes *CpA-Dcr2* and *CpA-R2d2*. Statistical analyses of survival were done with the Kaplan–Meier survival test through GraphPad Prism 8 software. *** *P <* 0.001. Three biological replicates were included with at least 50 female mosquitoes in each replicate. Data underlying this figure can be found in S2 Data. WT, wild type.

Multiple studies have shown that genetic engineering of mosquitoes and immune system activation is associated with fitness trade-offs [15,39–41]. To investigate the effects of transgenic Dcr2 and R2D2 expression and siRNA pathway activation on key fitness parameters, we compared the fecundity and hatch rate, pupation rate, adult size, and longevity of *CpA-Dcr2* and *CpA-R2d2* mosquitoes to those of their WT counterparts. The fecundity of both *CpA-Dcr2* and *CpA-R2d2* mosquitoes, as measured by the number of eggs laid after a naïve blood meal, was significantly lower than that of the WT controls (Fig 5A, Mann–Whitney test, ****P < 0.0001 and *P < 0.05, respectively), as was the egg hatch rate (Fig 5B, Mann–Whitney test, ****P < 0.0001 and *P < 0.05, respectively). These results indicate a general impairment of the reproductive rate and highlight the importance of DCR2, R2D2, and the siRNA pathway in mosquito reproduction. There was no significant difference in the pupation rate from day 6 to day 11 among the various mosquito cohorts (Fig 5C). The adult female and male body size, as determined by wing length, also did not differ between the transgenic and WT mosquitoes (Fig 5D). When mosquitoes were maintained on a 10% sucrose solution, there was no observable difference in life span between the transgenic and WT mosquitoes; this result was expected because transgene activation would need a blood meal (Fig 5E). After 1 blood meal, the *CpA-Dcr2* and *CpA-R2d2* females displayed a significantly longer life span than did the WT mosquitoes (Fig 5F, Kaplan–Meier survival analysis, ***P < 0.001), suggesting an effect that could be related to enhanced immune activity as also shown in Fig 4, which might have assisted the transgenic mosquitoes in suppressing the proliferation of harmful microbiota after the blood meal.

## Discussion

Although the function of the siRNA pathway has been studied in cultured cells, several published studies have linked the siRNA pathway to the restriction of DENV in *Ae*. *aegypti* mosquitoes, but the mechanism of siRNA-mediated viral restriction, and the translational potential of the control remain poorly understood. In the present study, overexpression of *Dcr2* and *R2d2* in the midgut through a blood meal–inducible promoter resulted in significant suppression of 3 human viral pathogens, DENV2, ZIKV, and CHIKV, both in the midgut tissue and systemically, with significant reduction of virus dissemination to the salivary glands. These results suggest that both DCR2 and R2D2 are restriction factors of the siRNA pathway, mediating an antiviral response that is important in controlling these viral infections. Furthermore, the broad-spectrum activity of this pathway against multiple viruses underscores its translational utility for disease control.

Previous studies with genetically engineered mosquitoes expressing specific small RNAs targeting either DENV or ZIKV resulted in significant suppression of virus [27,42–44], but the virus-specific activity has limited their application in vector control in areas where multiple arboviruses coexisting. Though resistance to both DENV and CHIKV was achieved previously through mosquito microRNA (miRNA) transgenesis [45], the rapid evolution of arboviruses in the field is likely to complicate this approach that is dependent on RNA sequence matches [46]. In contrast, our approach of overexpressing siRNA pathway factors is likely to represent a broad-spectrum antiviral approach that is more resilient to viral RNA sequence evolution. We have demonstrated that 1 transgenic line can block multiple arboviral transmissions.

In *Drosophila*, miRNAs that regulate the endogenous expression of mRNAs are sorted into a RISC containing AGO1. Specific isoforms of the dsRBP Loquacious (LOQS), LOQS-PA and -PB, interact with DCR1 to facilitate this process [47,48]. However, another isoform, LOQS-PD, appears to be involved in siRNA RISC loading, although it is unclear if the protein is involved in the antiviral (exogenous) pathway or only an alternate version of the siRNA pathway responsible for processing endogenous sources of dsRNA [49,50]. Furthermore, *Aedes* mosquitoes appear to lack an ortholog of the *Drosophila Loqs-PD*. Rather, another isoform, LOQS-PA, cooperates with R2D2 in the biogenesis of both endogenous and exogenous siRNAs [33]. More recently, a paralog of LOQS, LOQS2, is present in *Ae*. *aegypti* and *Ae*. *albopictus* but not in other mosquito species was postulated to be essential to control the systemic infection involved in the antiviral response through specific interactions with LOQS-PA and R2D2 [51]. Interestingly, *Loqs2* does not appear to be expressed in the midgut tissues of *Ae*. *aegypti*, and a transgenic mosquito expressing *Loqs2* under the control of the midgut-specific *carboxypeptidase* promoter exhibited significantly lower levels of DENV replication and dissemination, leading the authors of this study to suggest that LOQS2 activity is important for the restriction of systemic viral dissemination and replication in the mosquitoes [51]. Here, we showed that the production of ZIKV-siRNAs was significantly increased in *CpA-Dcr2* and *CpA-R2d2* transgenic mosquitoes, indicating that viral RNA was targeted by the RNAi machinery, either through direct DCR2-mediated degradation or indirectly enhanced DCR2 processing. Our results suggested that siRNA production from a viral substrate is effective in the midgut and could be enhanced through overexpressing factors of the siRNA pathway.

However, previous studies have shown that transgenic activation of the siRNA pathway through a construct with a double-stranded DENV template under the control of a blood meal–inducible midgut promoter results in significant suppression of DENV2 infection, suggesting that the biogenesis of endogenous siRNAs in the midgut may not be similarly defective [27,42]. Similarly, a transgenic *Ae*. *aegypti* mosquito line expressing a polycistronic cluster of engineered synthetic small RNAs targeting ZIKV in the midgut has shown a significant reduction in viral infection, dissemination, and transmission [43]. It is also interesting that the overexpression of both *Dcr2* and *R2d2* in the midgut was able to enhance the antiviral activity mediated by the siRNA pathway, despite the absence of LOQS2 in this tissue. That the overexpression of *R2d2* alone was able to mediate this enhanced activity is also somewhat surprising, as the protein is unstable in the absence of DCR2 [52], but this is consistent with previous observations in an *Ae*. *aegypti* cell line [33]. Studies in *Drosophila* have shown that R2D2 and LOQS-PD are able to decrease the concentrations of dsRNA that are effectively recognized and processed by DCR2 [53,54]. Perhaps LOQS2 serves a similar function in tissues other than the midgut, but this function can be rescued in the midgut through the transgenic overexpression of *Dcr2* or *R2d2*. However, regardless of the LOQS2 function, we showed through deep sequencing of small RNAs in ZIKV-infected transgenic mosquitoes that the overexpressed *Dcr2* and *R2d2* were in fact mediating the increased antiviral activity observed in the midgut through the siRNA pathway.

We used a traditional *Mos1 mariner* transposon-based transgenic approach that resulted in multiple transgenic lines with transgenes integrated into different chromosomal locations. Consequently, these lines varied in their ability to suppress virus infection as an outcome of positional effects; the chromosomal location of the transgene may influence its level of expression [55]. One advantage of a random integration-based transformation method is that it makes it possible to identify an optimal transgene integration site as we show in this study. Furthermore, *Mos1* has been proven as a useful vector for *Ae*. *aegypti* transformations that require a high degree of vector stability because of the rare germ-line transposition of *Mos1* in the presence of *Mos1* transposase [56]. In contrast to a transposon-based germ-line transformation approach, a site-specific docking line system, such as the ΦC31 site-directed recombination system, might allow better comparison between transgenes, since the transgenes would not differ because of positional effects [55].

Surprisingly, overexpression of *Dcr2* or *R2d2* resulted in broad transcriptomic changes involving genes belonging to diverse functional classes, and a significant number of these genes were specifically regulated by overexpression of either one of the 2 siRNA pathway factors, suggesting that DCR2 and R2D2 also perform other functions that may not be directly related to RNA degradation. Genes that were similarly regulated in recombinant *Dcr2*- and *R2d2*-expressing mosquitoes are likely to reflect the influence of the siRNA pathway on various biological systems, such as those related to development, cell proliferation, as well as immunity. As many as 62 regulated genes had putative roles in innate immunity, and many could be linked to the IMD and Toll pathways, suggesting a potential influence of the siRNA pathway on the mosquito’s antibacterial and antifungal defenses. Accordingly, we documented a significant suppression of the transgenic mosquito’s midgut microbiota and an increased resistance to systemic infections with both gram-negative and gram-positive bacteria, as well as with an entomopathogenic fungus. Indeed, the Toll pathway has been linked with defenses against gram-positive bacteria and fungi, while the IMD pathway has been linked to defenses against gram-negative bacteria [12,16,17]. However, the specific mechanisms and factors mediating this cross-talk between the siRNA pathway and the antimicrobial and antifungal innate immune pathways remain to be clarified through future studies. DCR2 has previously been shown to modulate the activity of the Toll pathway in *D*. *melanogaster* through a mechanism involving an interaction with the 3′ UTR of Toll transcripts, suggesting a pivotal role of the interaction between DCR2 and *Toll* mRNA in the Toll immune signaling [57,58]. Mosquito innate immunity, including both Toll and IMD pathways, is utilized to establish and promote *Wolbachia-Ae*. *aegypti* symbiosis that is being developed to control both DENV and ZIKV transmissions to humans [59]. The *Vago* gene, which was up-regulated in both *CpA-Dcr2*- and *CpA-R2d2*-transgenic mosquitoes, has been previously shown to link the siRNA pathway with the Jak-STAT pathway in *Culex* mosquitoes [60,61]. Comprehensive mapping of signaling pathway cross-talks in *Drosophila* cells has identified many transcriptional links between pathways, while the mechanisms remain to be characterized [62]. A recent study has identified a *Drosophila* viral suppressor of RNAi (VSR) interacting with long noncoding (lnc) RNA (VINR) that regulates a noncanonical antimicrobial pathway for the induction of AMPs. Flies absent of VINR showed a greater susceptibility to infections with virus and bacteria [63]. Several studies and reviews have addressed and highlighted immune modulatory of miRNAs and both insect- and pathogen-derived lncRNAs that are involved in pathogen-insect cross-talk [64–69].

The only negative fitness impact of *Dcr2* and *R2d2* overexpression that we observed was a decreased fecundity and egg hatch rate, and the transgenic and WT mosquitoes showed no difference in longevity when maintained on a sucrose solution. Trade-offs between immunity and reproduction are known to exist and could explain the slightly diminished fecundity we observed in these mosquitoes. Surprisingly, both *CpA-Dcr2* and *CpA-R2d2* transgenic mosquitoes displayed an increased longevity when given 1 naïve blood meal that would induce the transgenes. This effect could have resulted from a boosted immunity against both microbes as well as insect-specific viruses [31,70–72].

Although virus suppression in *CpA-Dcr2* and *CpA-R2d2* transgenic mosquitoes was highly significant, the leaky infection phenotype we observed warrants further study as part of the development of mosquitoes that are highly resistant to multiple viruses. Most likely, overexpression of siRNA pathway factors in the midgut tissue alone is not sufficient to completely suppress virus infection, and a few viral particles that will succeed in escaping this tissue are still able to cause systemic infection. Overexpression of multiple siRNA pathway factors, including AGO2 that has shown restriction of Mayaro virus [73], in multiple tissues could potentially result in a level of refractoriness that would have an epidemiologically significant impact on disease prevalence if WT mosquitoes would be replaced with transgenic. Furthermore, simultaneous transgenic expression of multiple orthogonal antiviral factors is likely to further potentiate virus blocking. Our study is therefore particularly timely given the current development of gene-drive technologies that can spread transgenes in mosquito populations even when the effector cargo would exert a certain fitness cost [74,75]. The recent frequent outbreaks caused by new, and reemerging, RNA viruses and the conserved function of siRNA-mediated anti-viral innate immune defenses have led to a regained momentum for RNAi therapy [76].

## Materials and methods

### Ethics statement

This study has been carried out in strict accordance with the recommendations in the Guide for the Care and Use of Laboratory Animals of the National Institutes of Health. Mice were used according to the approved animal protocol for rearing mosquitoes as a blood source for the maintenance of the mosquito colonies. The protocol (permit # MO15H144) was approved by the Animal Care and Use Committee of the Johns Hopkins University. Commercially obtained anonymous human blood type O+ and untyped human serum (Interstate Blood Bank, Inc.) were used for DENV and ZIKV infection assays in mosquitoes, and informed consent was therefore not required. Defibrinated sheep’s blood (Colorado Serum) was used for mosquito oral infection with CHIKV.

### Mosquito strains, rearing, and antibiotic treatment

*Ae*. *aegypti* Liverpool strain LVP-IB12 and transgenic mosquitoes generated for this study were maintained on 10% sucrose solution under standard insectary conditions at 27 ± 0.5°C and 75% to 80% humidity with a day:night light cycle of 14:10 h [37,38]. Mosquito rearing followed standard procedures established at the Johns Hopkins Insectary Core Facility, and colonies were maintained on Swiss Webster mice (Charles River Laboratories) anesthetized with ketamine.

To eliminate the natural microbiota from the mosquito midguts, antibiotics were applied according to a previously established protocol [36] with modifications. In brief, a single cohort of 120 adult female mosquitoes was collected immediately after eclosion and placed in a sterile cage. During the first week after eclosion, they were reared on a filter-sterilized 10% sucrose solution containing 100 mg gentamicin sulfate (Sigma-Alrich, Inc., St. Louis, USA) and 100 units/10 mg of penicillin–streptomycin (Thermo Fisher Scientific) per ml. The efficacy of the antibiotic treatment was measured by plating the midgut homogenates on an LB agar plate without antibiotics and by Giemsa staining of midgut homogenates [36]. One day prior to receiving a virus-infected blood meal, mosquitoes were provided with a simple sterile 10% sugar solution.

### Cloning of *Aedes Dcr2* and *R2d2* genes and the *AeCpA* promoter

The *Ae*. *aegypti Dcr2* and *R2d2* genes were PCR-amplified from *Ae*. *aegypti* cDNA using the primers Dcr2F and Dcr2R designed based on *Dcr2* (AY713296.1) covering the whole coding sequence (S1 Table). High-fidelity Long PCR Amplification Enzyme (LongAmp Taq DNA Polymerase, New England Biolabs, Ipswich, USA) was used with 35 cycles of: 30 s at 95°C, 30 s at 58°C, and 5 min at 68°C, with a final extension of 10 min at 68°C. The PCR product was purified and cloned into the ZeroBlunt II-TOPO vector (Thermo Fisher Scientific, Waltham, USA) with kanamycin as the selection marker, followed by sequencing verification through multiple internal primers (S1 Table). Similarly, the R2d2F and R2d2R primers were used to amplify the CDS of the *R2d2* gene (KJ598053.1). The protocol was similar to the above, but with a shortened extension time (1 min). The carboxypeptidase A promoter (*pAeCpA*) fragment (1.6 kbp) was amplified using AeCpAF and AeCpAR primers from genomic DNA (gDNA) based on the upstream sequences of the carboxypeptidase A gene (*AeCpA*) (AF165923) (S1 Table). The PCR protocol was 35 cycles of 30 s at 95°C, 30 s at 54°C, and 2 min at 72°C, with a final extension of 5 min at 72°C, followed by cloning into the pGEM-T Easy (Promega, Madison, USA) and verification by sequencing.

### pBluescript-based constructs of *AeCpA-Dcr2* and *AeCpA-R2d2*

The pBluescript II SK(-) (pBSK)-based *pAeCpBvDome-TrypT* plasmid (previously generated in the lab containing the *AeCpB-V* promoter sequence [15]) was double-digested with *Xh*oI and *Hind*III to excise the *AeCpB*-V promoter and substituted with the *AeCpA* promoter. In brief, the *AeCpA* promoter was amplified using the respective primers (S1 Table) to add different restriction sites to ensure ease in downstream subcloning. To generate *AeCpA-R2d2-TrypT-pBSK*, the *Dome* gene on the *AeCpBvDOME-TrypT-pBSK* plasmid was swapped with *R2d2*. The primers R2D2F_NarI_PBS and R2D2R_XbaI_PBS were used to place the *R2d2* in the pBSK construct (S1 Table). As described above, the 1.6-kb PCR-amplified *AeCpA* promoter fragment derived from the pGEM-T Easy vector was subcloned at the upstream of the *R2d2* to replace the *AeCpBv* promoter; for this purpose, AeCpAF_Xho1_Fse_PBS and AeCpAR_Nar_PBS were used (S1 Table). The construct *AeCpA-Dcr2-TrypT-pBSK* was generated in a similar manner using AeCpAF_Xho1_Fse_PBS and AeCpAR_Avr_PBS as the forward and reverse primers, respectively. The positive clones were verified by sequencing and used for downstream subcloning experiments.

### Construction of Gateway destination clones

Using a cloning method based on digestion and T4 ligation of the *Dcr2* gene plus the promoter and terminator sequences (*AeCpA-Dcr2-TrypT*, >7 kb in length) into *Mos1 mariner* vectors [56,77] at the *Fse*I site proved laborious, and after many failures with this traditional cloning method, we adopted the use of an easier and much more efficient system, the Gateway cloning system (Thermo Fisher Scientific). Beforehand, we modified *pMos1* (*Mos1 mariner*) vectors to ensure the proper and efficient cloning of large gene constructs. We amplified the Gateway cassette sequence from the *pMinos-att* vector (kindly provided by Dr. Yoshito) [78] containing the *ccd*B gene, the chloramphenicol resistance gene, and *attR1* and *attR2* sequences flanked at the 5′ and 3′ ends of the Gateway cassette by using the primers listed in S1 Table. The PCR-amplified fragments flanked with *attR* sites were cloned into the unique *Fse*I site of the *pMos1* vector (*pMos1-3xP3-DsRed* and *pMos1-3xP3-eCFP*) to generate destination *pMos1-attR* vectors. After overnight digestion with *Fse*I of the *pMos1* vectors, CIP was used to treat the blunt-ended digested vectors to avoid self-ligation. The ligated constructs were transformed into *ccd*B-resistant *E*. *coli* competent cells, *ccd*B Survival 2 T1R (Thermo Fisher Scientific, Waltham, USA), derived from the TOP10 strain. The positive clones were selected using LB agar plates containing chloramphenicol (25 μg/ml) and ampicillin (100 μg/uL) antibiotics, followed by plasmid miniprep, and sequencing to select the appropriate clones.

### Construction of Gateway entry and expression clones

The PCR primers (S1 Table), with *CACC* nucleotides at the 5′ ends, were used to PCR-amplify large gene fragments from pBSK clones, followed by cloning of these gene fragments into the pENTR/D-TOPO cloning vector (Thermo Fisher Scientific, Waltham, USA). Gene-of-interest gene fragments were PCR-amplified from *AeCpA-Dcr2-TrypT-pBSK* and *AeCpA-R2D2-TrypT-pBSK* using the primers pEntrCpA_F and pEntrTryp_R. Ligations were set up using 50 to 100 ng of blunt-ended PCR product and 20 ng of pENTR-TOPO for 15 to 30 min at room temperature, followed by transformation into Top10 *E*. *coli-*competent cells (Thermo Fisher Scientific, Waltham, USA). The screening of positive clones that grew on the LB agar plates containing 50 μg/ml kanamycin was done by colony PCR, followed by plasmid mini-prep, then RE digestion, and sequencing. An LR recombination reaction using Gateway LR Clonase II enzyme mix was used to generate the expression vectors using the corresponding *pEntr* vectors and destination *pMos1-attR* vectors (*pMos1-3xP3-eCFP-attR*, *pMos1-3xP3-dsRed-attR*). LR reaction mixtures were transformed into *E*. *coli* DH5α competent cells and spread on LB agar plates (containing 100 μg/ml ampicillin) for the selection of positive recombinant expression clones. Two expression vectors, *pMos1-AeCpA-Dcr2-TrypT-3xP3-dsRed* and *pMos1-AeCpA-R2d2-TrypT-3xP3-eCFP*, were verified by sequencing (plasmid maps and sequences are available in S1 Data).

### Embryonic microinjection and generation of transgenic *Aedes* mosquitoes

For the germ-line transformation, the donor expression plasmids generated above and helper plasmid pKhsp82MOS were prepared using the Endofree Maxi Prep kit (Qiagen, Germantown, USA) and resuspended in 1× microinjection buffer (5 mM KCl, 0.1 mM sodium phosphate (pH 6.8)) at a ratio of 300 ng/μL donor versus 500 ng/μL helper plasmid according to published methods [39,40,79,80]. Three days post-blood feeding of females, embryos were collected 45 min before microinjection. Embryonic microinjection was performed using an Eppendorf FemtoJect Express and quartz needles. Microinjected eggs were washed carefully with distilled water using a water bottle to remove as much as possible of the halocarbon oil 27; these eggs were then allowed to remain on the slide, submerged in a small container of distilled water for 30 min, before being brushed off from the slides onto wet paper towels. To hatch these eggs, these injected embryos on the wet paper towels were transferred, 4 days later, to 1 L of autoclaved hatching broth (1 TetraMin Tropical tablet in 1 L of distilled water). The hatched G_0_ larvae were then transferred to clean water and fed with TetraMin Tropical tablets according to standard mosquito rearing protocols. Hatched larvae (G_0_ generation) were screened with a fluorescence microscope, then sexed at the pupal stage, and crossed separately with the opposite sex of WT mosquitoes at a ratio of 3 females to 1 male. For the *CpA-Dcr2* transgenic line, approximately 2,100 to 2,200 embryos were injected, and the 600 larval survivors that hatched (30% hatching rate) and developed into adults were outcrossed to WT mosquitoes in several cohorts. For the *CpA-R2d2* lines, approximately 1,200 embryos were injected, and the 400 larval survivors that hatched (30% to 35% hatching rate) were outcrossed with WT mosquitoes in the same manner. The G1 larvae from several successive gonotrophic cycles were examined for the presence of blue (eCFP) and red (DsRed) eye colors under a dissection fluorescence stereomicroscope (Olympus America, Center Valley, USA). Fluorescent larvae were carefully isolated and allowed to develop into adults. After 3 generations of outcrossing with WT mosquitoes, the heterozygous mosquitoes were inbred for at least another 8 generations to generate homozygous *CpA-Dcr2* and *CpA-R2d2* lines for further characterization. Inbred crosses were made at the same ratio of 3 females to 1 male (groups of 3 to 15 males crossed with 10 to 45 females), which finally produced 2 *CpA-Dcr2* and 7 *CpA-R2d2* lines.

### Virus propagation in cell cultures and oral viral infections in *Ae*. *aegypti*

DENV serotype 2 New Guinea C strain (DENV2), ZIKV strain FSS 13025, and CHIKV strain 99659 were used in this study. DENV2 and ZIKV were cultured in *Aedes albopictus* C6/36 cells (ATCC CRL-1660), and viral stocks were prepared as previously described in [5,15,81–84]. In brief, C6/36 cells were cultured in MEM medium (Gibco, Thermo Fisher Scientific, Waltham, MA, USA) supplemented with 10% heat-inactivated fetal bovine serum (FBS), 1% penicillin–streptomycin, and 1% non-essential amino acids and maintained in a tissue culture incubator at 32°C and 5% CO_2_. Baby hamster kidney strain 21 (BHK-21, ATCC CCL-10) and green monkey kidney (Vero) (ATCC) cells were maintained at 37°C and 5% CO_2_ in the DMEM medium (Gibco, Thermo Fisher Scientific, Waltham, USA) supplemented with 10% FBS, 1% penicillin–streptomycin, and 5 μg/ml Plasmocin (InvivoGen, San Diego, USA). For DENV2 and ZIKV viral stock preparation, C6/36 cells grown to 80% confluence were infected with ZIKV and DENV2 at a multiplicity of infection (MOI) of 10 and incubated at 32°C and 5% CO_2_ for 6 days or 5 days for DENV2 or ZIKV, respectively. The virus was harvested by 3 freeze–thaw cycles using dry ice and a water bath (37°C), followed by centrifugation at 2,000 rpm for 10 min at 4°C. The supernatant from this cell lysis was mixed with the original cell culture supernatant to yield the final viral stock. Viral stocks were aliquoted and stored at −80°C for long-term storage. CHIKV was amplified in green monkey kidney (Vero) (ATCC) cells at an MOI of 0.01 and harvested approximately 36 h later. Viral stock titration was done by plaque assay.

Mosquitoes were orally infected with DENV2 or ZIKV via artificial glass membrane feeders as previously described [5,81]. Blood meal viral titration was done together with the infected mosquito samples by plaque assay. Three to 5-day-old mosquitoes were fed a blood meal containing approximately 2.5 × 10^6^ PFU/ml of CHIKV [83,84]. The virus prepared in Vero cell culture was mixed 1:1 with defibrinated sheep’s blood (Colorado Serum, Denver, USA). Infectious blood meals were offered through an artificial membrane using the Hemotek feeding system (Discovery Workshops, Accrington, United Kingdom). A portion of each blood meal was frozen, and back titers were determined by plaque assay on Vero E6 (ATCC) cells at 37°C. Mosquitoes were starved for 24 h prior to being offered the blood meal and were allowed for feed for approximately 30 min. Fully engorged mosquitos were sorted into soup cups, with no more than 20 individuals per cup. This propagation yielded virus titers of 1.0 × 10^6–7^ for DENV2, 1.0 × 10^7–9^ for ZIKV, and 1.0 × 10^6–7^ PFU/ml for CHIKV. Each experiment was performed in at least 3 biological replicates, as indicated.

### Plaque assays for viral titration

BHK-21 and Vero cells were maintained on DMEM medium supplemented with 10% FBS, 1% penicillin–streptomycin, and 5 μg/ml Plasmocin (InvivoGen) at 37°C and 5% CO_2_. DENV2- and ZIKV-infected mosquito samples or viral stocks were titrated in the BHK-21 cell culture, and CHIKV viral stocks were titrated on Vero cell monolayers. All infection procedures were performed under BSL2 conditions for DENV2 and ZIKV and BSL3 conditions for CHIKV (Dr. Myles’ lab). For DENV2- and ZIKV-infected mosquito tissues, plaque assays were used to determine infection prevalence and viral titer. In brief, mosquito midguts, carcasses, or salivary glands were collected at 7 and 14 days post-infectious blood meal (PIBM) in 150 μl of complete DMEM medium with glass beads. A single mosquito was used for 1 sample. A Bullet Blender (Next Advance, Troy, USA) was used to homogenize the tissue samples, and serial dilutions were prepared with DMEM complete medium. The BHK-21 cells were split to give a 1:10 dilution and grown on 24-well plates to 80% confluence 1 to 2 days before the plaque assays. After serial dilution, the mosquito tissue or viral stock samples (100 μl each) were added to the BHK-21 cells, followed by incubation at room temperature for 15 min on a rocking shaker (VWR International LLC) and subsequent incubation at 37°C with 5% CO_2_ in a cell incubator (Thermo Fisher Scientific, Waltham, USA) for another 45 min. The 24-well plates with infected BHK-21 cells were overlaid with 1 ml of 0.8% methylcellulose in complete DMEM medium with 2% FBS and incubated for 5 to 6 days in a cell culture incubator (37°C and 5% CO_2_). Plaques were fixed and developed with staining reagent (1% crystal violet in 1,1 methanol/acetone solution) at room temperature for 1 h. Plates were rinsed with distilled water and air-dried, and plaques were counted and multiplied by the corresponding dilution factors to calculate the plaque-forming units (PFUs) per sample. Three biological replicates were performed, with 18 to 36 mosquitoes per replicate.

### Analysis of CHIKV viral infection/dissemination by CPE assay

To check the infection prevalence of CHIKV in the individual mosquitoes, at 7 dpi, the mosquitoes were cold-anesthetized and decapitated. Head and bodies were placed into corresponding 1.7-ml tubes and stored at −80°C until processed. CHIKV-infected mosquito bodies or heads were removed from the −80°C freezer in small groups and individually triturated in 300 μl of DMEM containing 10% heat-inactivated FBS, 1% penicillin–streptomycin, 1 μg/ml fungizone (amphotericin B), and 50 μg/ml gentamicin. Homogenates were filtered through 0.22-μm syringe filters (Corning, NY, USA) into fresh screw cap tubes. The C6/36 cell suspension and 100 μl of the filtrate were added to individual wells of a 96-well plate and placed at 28°C with 5% CO_2_. After a 48-h incubation, the BHK-21 cell suspension and 75 μl of supernatant from each well were added to a new 96-well plate and placed at 37°C with 5% CO_2_. Cells were scored for the presence of CPE on days 2 to 4 [84]. Three biological replicates were performed, with 29 to 40 mosquitoes per replicate.

### RT-PCR-based quantification of viral replication of CHIKV

To check the viral titers of the CHIKV-infected mosquito samples, a subset of mosquitoes were tested for disseminated infection as described above. Midguts, salivary glands, and carcasses from positive mosquitoes were collected in pools of 5 per biological replicate. Total RNA was extracted from the pools using TRI Reagent RT (Molecular Research Center Inc., Cincinnati, USA) according to the manufacturer’s protocol. Approximately 300 ng of total RNA were reverse-transcribed using SuperScript IV reverse transcriptase (Thermo Fisher Scientific, Waltham, USA) according to the manufacturer’s protocol. qRT-PCR was performed using the probe and primers described in [83]. The numbers of CHIKV positive-strand NSP1 copies were calculated from the standard curve.

### Immunofluorescence confocal microscopy

Immunostaining for confocal microscopy was performed as previously described [85,86] with modifications. The midguts of DENV2-infected WT, transgenic *CpA-Dcr2*, and *CpA-R2d2* mosquitoes were dissected at 7 dpi in 1% paraformaldehyde solution and fixed in 4% paraformaldehyde overnight (approximately 16 h). About 10 midguts were prepared for 1 treatment and were thoroughly washed with 1× PBS buffer to remove the paraformaldehyde, followed by incubation with 10% goat serum (Invitrogen, Thermo Fisher Scientific, Waltham, USA) to block background staining. After blocking at room temperature for 2 h, the midguts were removed and stained with mouse anti-flavivirus envelope protein antibody 4G2 (ATCC) at a 1:400 dilution for 24 h at 4°C in the refrigerator. AlexaFluor 568 goat anti-mouse IgG (Invitrogen, Thermo Fisher Scientific, Waltham, USA) was used as the secondary antibody at a 1:1,000 dilution. Beta-actin was stained with AlexaFluor 488 Phalloidin (Invitrogen, Thermo Fisher Scientific, Waltham, USA), and nuclei were stained with DAPI. Samples were mounted with ProLong Gold antifade reagent (Invitrogen, Thermo Fisher Scientific, Waltham, USA), and 0.2- to 1-mm optical sections were cut and examined with a Zeiss LSM 710 confocal microscope. The confocal microscopy settings were kept the same across all slides for comparison purposes, and the DAPI staining intensity of each treatment was used for standardization. Projections of the stacks of the images were produced through Zeiss (Jena, Germany) Zen and Fiji ImageJ software.

### gDNA extraction, RNA isolation, and quantitative real-time PCR (qRT-PCR)

A Qiagen DNeasy Tissue kit (Qiagen, Germantown, USA) was used to extract gDNA from whole WT, *CpA-Dcr2*, and *CpA-R2d2* transgenic mosquitoes. About 10 ng of gDNA was used for conventional PCR to confirm the insertion of the transgenes (*CpA-Dcr2*, *CpA-R2d2*) in the transgenic mosquitoes, using the corresponding primers listed in S1 Table.

Mosquito total RNA was extracted using TRIzol Reagent (Invitrogen, Thermo Fisher Scientific, Waltham, USA) according to the manufacturer’s instructions. The RNA concentration was measured with a NanoDrop Spectrophotometer. Genomic DNA was removed by treatment with Turbo DNA Free (Ambion, Austin, USA), and cDNA was synthesized using approximately 1 μg total RNA and an M-MLV Reverse Transcriptase kit (Promega, Madison, USA) according to the manufacturer’s instructions. The resulting cDNA was diluted from 20 μl to 50 μl, and 1 μl of cDNA was used as a template for qRT-PCR analysis.

The qRT-PCR assays were done according to [39] with the primers listed in S1 Table. The *Ae*. *aegypti* housekeeping *Rps*17 was used for normalization. The SYBR Green PCR Master Mix (Applied Biosystems, Thermo Fisher Scientific, USA) and ABI StepOnePlus Real-time PCR system with ABI StepOne Software were used according to the manufacturer’s protocol. The fold-change in the gene expression was calculated according to the standard E^ΔΔCt^ method [87] when both the primer efficiencies of the gene of interest and the *Rps*17 gene are equal. The primer efficiencies were determined as described in [36]. The relative abundance of ZIKV RNA, as a measure of viral load, in the mosquitoes at 2 or 4 dpi was measured through qRT-PCR with specific ZIKV primers (S1 Table), and the *AeRps7* gene was used as internal control.

### Inverse PCR

Inverse PCR was done essentially according to a published protocol [88] with modifications. Genomic DNA was digested overnight with Sau3AI and CviAII (New England Biolabs, Ipswich, MA, USA). The digested DNA was purified (Qiagen PCR purification kit) and ligated with T4 DNA ligase (New England Biolabs, Ipswich, MA, USA) and used as a template for the following PCR reaction: 98°C for 3 min., 37 cycles of 98°C for 30 s, 58°C for 45 s, 72°C for 3 min, and 1 cycle of 72°C for 5 min, using the primers listed in S1 Table. Amplification products were gel extracted with 1% agarose gel and sequenced. VectorBase (http://www.vectorbase.org) was searched for sequences corresponding to the junctions between transposon landing sites on *Ae*. *aegypti* genome and transposon Mos1 left arms using the BLASTn tool.

### RNA-seq library preparation, sequencing, and bioinformatics analysis

At 1 day PBM, total RNA was extracted from 8 midguts of transgenic *CpA-Dcr2* and *CpA-R2d2* or WT mosquitoes per sample using TRIzol reagent (Invitrogen, Thermo Fisher Scientific, USA); 3 independent biological replicates were included for each treatment. Both the sequencing library construction and sequencing were performed by Novogene (Beijing, China). An Illumina sequencing library was prepared for each RNA sample according to the manufacturer’s instructions and sequenced on the Illumina platform with paired-end 150 bp (PE 150). Final sequencing data were deposited into NCBI’s Sequence Read Archive (SRA: PRJNA838236). Reads containing adapters, poly-N, and low-quality reads were removed from raw data in FASTQ format, and final cleaned reads were aligned to the *Ae*. *aegypti* genome (AaegL4.1). FeatureCounts (a software program) was used to quantify transcript abundance in each sample using the gene annotation AaegL4.1 obtained from VectorBase, and the number of fragments per kb of transcript per million mapped reads (FPKM) for each gene was calculated, based on the length of the gene and the read count mapped to the gene. Genes with a *p*-value <0.05 and -fold changes in transcript abundance on a log2 scale >0.75 (equivalent to >1.68 fold-changes) obtained by DESeq2 were considered DEGs between transgenic mosquitoes and WT controls. GO enrichment analysis was performed with the GOstats package in R (http://www.bioconductor.org/packages/release/bioc/html/GOstats.html). Overrepresentation of the gene functional category based on previous classification [15] was performed using the hypergeometric test with the phyper package in R [35]. Overrepresentation of a DE gene functional category was based on previous classification [15]. Venn diagrams were generated using Venny 2.1 (http://bioinfogp.cnb.csic.es/tools/venny/index.html).

### Small RNA library construction and bioinformatic analyses

Total RNA of the midguts at various time points post infectious blood meal prepared with TRIzol reagent (Invitrogen, Thermo Fisher Scientific, USA) was used as the input for library preparation, utilizing the SMARTer smRNA-Seq Kit for Illumina (Clontech/TaKaRa, USA). The small RNA sequencing was performed at the Johns Hopkins Transcriptomics and Deep Sequencing Core Facility. To remove the sequences smaller than 18 nt after sequencing, the raw sequenced reads were subjected to Cutadapt (v2.8) for raw adaptor trimming. The remaining sequences were mapped to the *A*. *aegypti* reference genome, the ZIKV genome using Bowtie [89], allowing no mismatches and discarding reads mapping on multiple regions. Size profiles of small RNAs matching the ZIKV genome and 5′ nt frequency were calculated using custom-built BioPerl library and R scripts. Plots were prepared in Microsoft Excel. Transcriptome analysis of public data was performed using raw reads as input for RNA-seq STAR followed by normalization through edgeR (a Bioconductor package).

### Systemic or oral bacterial challenge survival assays

Standard laboratory strains of *E*. *coli* (DH5α) and *S*. *aureus* were used to represent gram-negative and gram-positive bacteria, respectively, and systemic bacterial challenge assays were carried out essentially according to established protocols [36,39]. Bacteria were cultured in Luria-Bertani (LB) medium at 37°C at 250 rpm overnight (approximately 16 h,) followed by 2 washes with 1× PBS buffer. *E*. *coli* and *S*. *aureus* cell pellets were resuspended in 1× PBS to OD_600_ = 2.0 or = 3.0, respectively. Before the bacterial challenge, transgenic or control WT mosquitoes were given a naïve blood meal on mice to activate the expression of either *Dcr2* or *R2d2* in the midgut, then 69 nl of bacterial suspension was injected into the thorax of each cold-anesthetized mosquito at 24 h PBM with a nano-injector (NanojectII, Drummond Company, Inc., Birmingham, USA). For the non-bacterially challenged negative controls, both transgenic and WT mosquitoes were injected with 1× PBS buffer. Thirty blood-fed females were injected with either 1× PBS or bacteria, and at least 3 replicates were included in all experiments. Oral bacterial challenges were applied through 3% sugar feeding, the bacteria were resuspended in the final concentration of OD_600_ = 4.0 in a sterile 3% sucrose solution and delivered to the mosquitoes through a piece of cotton ball. The significance of transgene *Dcr2* or *R2d2* expression for the mosquitoes’ susceptibility to the bacterial challenge was measured using Kaplan–Meier survival analysis with a log-rank test, using GraphPad Prism 8 software [36].

### Quantification of proliferated midgut microbiota through colony counting and 16s qPCR

Isolation and enumeration of the midgut microbiota from WT, *CpA-Dcr2*, and *CpA-R2d2* mosquitoes in terms of CFUs were conducted as previously described [36,39]. All mosquitoes were age matched and 5- to 6-day-old mosquitoes were given a naïve blood meal on mice; the engorged mosquitoes were then separated from the unfed mosquitoes and kept in a separate cage within the standard insectary chamber for the remainder of the experiment. On the day of the blood feeding, the unfed mosquitoes were sorted as the samples for the time 0 h group, and the rest of the mosquitoes were used for the PBM analyses at the corresponding time points of 24, 48, and 72 h. Each cohort of 12 mosquitoes from each group was surface sterilized before the midguts were dissected out; this surface sterilization was performed by incubating the mosquitoes in 70% ethanol for 2 min, followed by 2 rinses in sterile 1× PBS. Treated mosquitoes were placed on a plain LB agar plate for 1 min prior to incubation at RT for 48 h to determine bacterial growth and thereby the efficacy of sterilization. Each midgut was dissected with forceps in sterile PBS, then placed in 200 μl of PBS, and homogenized for 30 s. About 10-fold serial dilutions of the homogenates were prepared, and 100 μl of the 10^−2^ and 10^−4^ dilutions were plated on plain LB agar and incubated at ambient temperature for 3 to 4 days before the colonies were counted. Each experiment was performed using a single midgut, and the results are given as the mean CFU value from 12 samples. Total bacterial abundance was also compared through qRT-PCR on the 16s RNA, including both culturable and unculturable bacteria, according to an established protocol [90].

### Wing length, life span, fecundity, and egg hatchability

Adult wing length (in both males and females) was used as a surrogate measurement for mosquito size. Mosquitoes were anesthetized on ice and kept on a cold plate for wing length measurement. Wing length was measured manually from the distal end of the alula to the tip of the wing (without the hairy fringe) through a microscope objective containing a scale bar calibrated to a 0.1-mm stage, without taking pictures or using software [90].

To measure the life span of the WT and transgenic mosquito lines, within 12 h of emergence the adult mosquitoes were placed into 16-oz soup cups covered with a net. A small cotton ball was placed on top of the net and was constantly impregnated with a 10% sucrose solution. They were held there until all the mosquitoes in that cup had died, and the number of dead mosquitoes in the cup was recorded and the dead mosquitoes were removed every other day [39,90]. To determine the life span of the mosquitoes fed upon a blood meal, the mosquitoes were offered a blood meal on mice 5 to 6 days after emergence, and only mosquitoes taking a blood meal were retained for the rest of the study. The survival percentage represents the mean survival percentage for all 3 biological replicates of 30 mosquitoes each. Statistical significance was determined by Kaplan–Meier survival analysis with pooled data from 3 replicates by using GraphPad Prism 8 software, and *p*-values were determined by the Wilcoxon test as described in [39,41].

For the fecundity assay, approximately 50 seven-day-old adult female WT, *CpA-Dcr2*, and *CpA-R2d2* mosquitoes were allowed firstly to feed on mice. The mosquitoes were anesthetized on ice immediately following the blood meal, and all non-engorged mosquitoes were discarded. At 3 days after blood feeding, the female mosquitoes were separated into individual vials (50-ml Falcon tubes) containing moist filter paper with 2 ml of water on the bottom and allowed to oviposit, and the number of eggs laid by each female was recorded at 4 to 5 days after the blood feeding using light microscopy. The females that died before laying eggs were excluded from the assays. After each count, the filter paper with the eggs was submerged in larval rearing water in a 5-oz plastic cup to allow the eggs to hatch. First- and second-instar larvae were counted under a light microscope. Statistical significance was determined using the Mann–Whitney test with GraphPad Prism 8 software. The control mosquitoes were reared under the same conditions as the transgenic mosquitoes.

### Statistical analysis

Infection intensity, wing length, fecundity, and hatch rate were assessed by the Mann–Whitney test and infection prevalence by the Fisher’s exact test with GraphPad Prism 8 software. The statistical significance of mosquito survival rate was determined by Kaplan–Meier survival analysis with pooled data from 3 replicates by using GraphPad Prism 8 software, and *p*-values were determined by the Wilcoxon test. The significance of mosquitoes’ susceptibility to the bacterial challenge was measured using Kaplan–Meier survival analysis with a log-rank test, using GraphPad Prism 8 software [36]. The Student *t* test was used for the determination of the significance of fold changes in gene expression and bacterial CFU loads in the mosquito midguts. *P* < 0.05 was considered significant.

## Supporting information

S1 Fig DENV2 infection titers and prevalence at 7 dpi in the midguts of different heterozygous transgenic lines at G_4_ generation. Both viral titers and the percentage of infected mosquitoes (prevalence) from *CpA-R2d2* transgenic lines (L1–L7) and *CpA-Dcr2* lines (L1–L2) are presented. Plaque assays were used to determine viral loads and infection prevalence. Graphpad Prism 8 software was used to compare median virus titers through the Mann–Whitney test. Statistical analyses comparing infection prevalence values were made using the Fisher’s exact test. * *P <* 0.05, ** *P <* 0.01, *** *P <* 0.001, compared to WT. Data underlying this figure can be found in S2 Data. DENV2, DENV serotype 2; dpi, days post-infection; WT, wild type.
(DOCX)

S2 Fig ZIKV2 infection titers and prevalence at 7 dpi in the midguts of different heterozygous transgenic lines at G_4_ generation. Both viral titers and the percentage of infected mosquitoes (prevalence) from *CpA-R2d2* transgenic lines (L1–L7) and *CpA-Dcr2* lines (L1–L2) are presented. Plaque assays were used to determine viral loads and infection prevalence. Graphpad Prism 8 software was used to compare median virus titers through the Mann–Whitney test. Statistical analyses comparing infection prevalence values were made using the Fisher’s exact test. * *P <* 0.05, ** *P <* 0.01, *** *P <* 0.001, compared to WT. Data underlying this figure can be found in S2 Data. dpi, days post-infection; WT, wild type; ZIKV, Zika virus.
(DOCX)

S3 Fig The total bacterial loads of the midgut microbiota of female transgenic and WT control mosquitoes at 24, 48, and 72 h PBM (mean ± SEM) measured by qRT-PCR of *16s* bacterial ribosomal gene. Log2 transformed fold-change is presented. *Ae*. *aegypti Rps*17 gene was used for the normalization. Data underlying this figure can be found in S2 Data. PBM, post-blood meal; qRT-PCR, quantitative real-time PCR; WT, wild type.
(DOCX)

S4 Fig The survival rate of transgenic mosquitoes after oral challenge with either gram-positive (*S*. *aureus*: 800,000 CFU) or gram-negative (*E*. *coli*: 800,000 CFU) bacteria at 7 dpi (**A, B**). All groups of the mosquitoes in (B) were given a naïve BM to up-regulate the expression of the transgenes, while no BMs were given to mosquitoes in (A). The significance of the survival rates was determined by Kaplan–Meier survival analysis from 3 biological replicates (ns: not significant). Data underlying this figure can be found in S2 Data. BM, blood-meal; CFU, colony-forming unit; dpi, days post-infection.
(DOCX)

S5 Fig qRT-PCR expression profiling of a panel of antimicrobial and immune genes in the WT and transgenic *CpA-Dcr2* mosquito carcasses at 0 h (before blood meal), 24 h, 48 h, and 72 h PBM. The WT carcasses were used as the control, and the *AeRps17* gene was used as an internal control for normalization. Data underlying this figure can be found in S2 Data. PBM, post-blood meal; qRT-PCR, quantitative real-time PCR; WT, wild type.
(DOCX)

S1 Table Primers used for the generation of constructs for embryo microinjections to generate transgenic mosquitoes, verification of transgenes on the transgenic mosquito chromosome, and qRT-PCR primers. (DOCX)

S2 Table DNA sequence obtained by inverse PCR flanking the transposon Mos1 random insertions from both *CpA-Dcr2* and *CpA-R2d2* transgenic mosquitoes. Inverse PCR was done using primers (MLF1 and MLR1) in **S1 Table.**
(DOCX)

S3 Table Enriched immune genes related to the immune pathways in the *CpA-Dcr2* and *CpA-R2d2* transgenic mosquitoes at 24 h post blood meal (detailed gene list of Fig 3G and 3H). (DOCX)

S1 Data The maps and the sequences of the plasmids used for embryo microinjections to generate both *CpA-Dcr2* and *CpA-R2D2* transgenic mosquitoes. (DOCX)

S2 Data An excel file with the detailed individual numerical values that underlie the summary data displayed in the figure panels: Figs 2A–2I, 3A–3F, 3H, 3I, 4A–4H, 5A–5F, S1, S2, S3, S4A, S4B, and S5. (XLSX)

S3 Data RNA seq details. (XLSX)

S1 Raw Images The gel picture of Fig 1D. (PDF)

## Abbreviations

AMP: antimicrobial peptide
CHIKV: chikungunya virus
CPE: cytopathic effect
CFU: colony-forming unit
DEG: differentially expressed gene
DENV: dengue virus
DENV2: DENV serotype 2
dpi: days post-infection
dsRBP: double-stranded RNA binding protein
dsRNA: double-stranded RNA
FBS: fetal bovine serum
gDNA: genomic DNA
GO: gene ontology
IFA: immunofluorescence microscopy assay
miRNA: microRNA
MOI: multiplicity of infection
PAMP: pathogen-associated molecular pattern
PBM: post-blood meal
PFU: plaque-forming unit
PIBM: post-infectious blood meal
PRR: pattern recognition receptor
qRT-PCR: quantitative real-time PCR
RISC: RNA-induced-silencing-complex
siRNA: small interfering RNA
WT: wild type
ZIKV: Zika virus
