# Correction: The *Aedes aegypti* siRNA pathway mediates broad-spectrum defense against human pathogenic viruses and modulates antibacterial and antifungal defenses

**DOI:** 10.1371/journal.pbio.3001722

**Published:** 2022-07-07

**Authors:** The PLOS Biology Staff

## Notice of republication

This article was republished on June 22, 2022 to remove an unneeded Abstract Teaser. The publisher apologizes for the error. Please download this article again to view the correct version.
