# Supplementary material for: The Aedes aegypti siRNA pathway mediates broad-spectrum defense against human pathogenic viruses and modulates antibacterial and antifungal defenses
Source: PLoS Biol. 2022 Jun 9;20(6):e3001668. doi: 10.1371/journal.pbio.3001668 (PMC9182253; doi:10.1371/journal.pbio.3001668)
Supplement: S2 Fig — Both viral titers and the percentage of infected mosquitoes (prevalence) from CpA-R2d2 transgenic lines (L1–L7) and CpA-Dcr2 lines (L1–L2) are presented. Plaque assays were used to determine viral loads and infection prevalence. Graphpad Prism 8 software was used to compare median virus titers through the Mann–Whitney test. Statistical analyses comparing infection prevalence values were made using the Fisher’s exact test. * P < 0.05, ** P < 0.01, *** P < 0.001, compared to WT. Data underlying this figure can be found in S2 Data. dpi, days post-infection; WT, wild type; ZIKV, Zika virus. (DOCX) [file pbio.3001668.s002.docx]

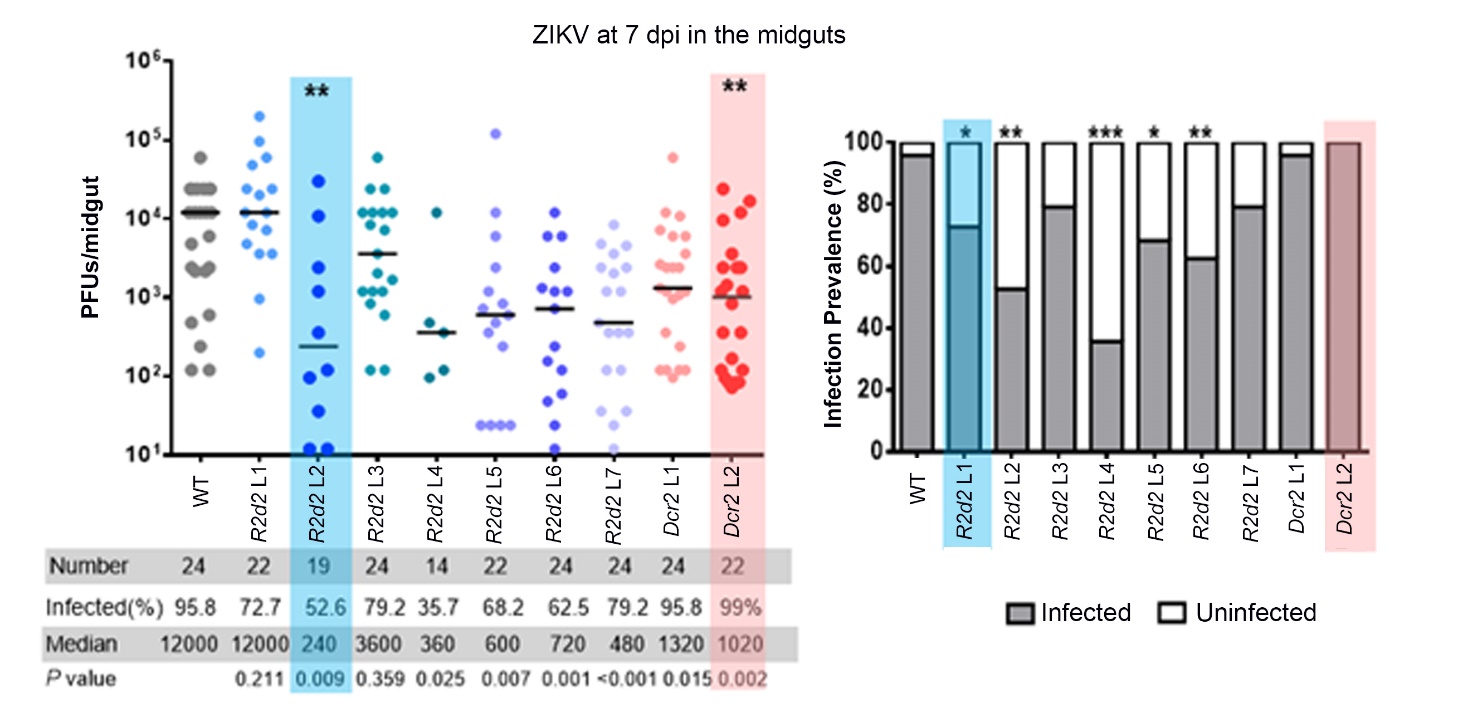


**S2 Fig.** ZIKV2 infection titers and prevalence at 7dpi in the midguts of different heterozygous transgenic lines at G_4_ generation. Both viral titers and the percentage of infected mosquitoes (prevalence) from *CpA-R2d2* transgenic lines (L1-L7) and *CpA-Dcr2* lines (L1-L2) are presented. Plaque assays were used to determine viral loads and infection prevalence. Graphpad Prism 8 software was used to compare median virus titers through either the Mann-Whitney test or Kruskal-Wallis test with Dunn’s post-test. Statistical analyses comparing infection prevalence values were made using the Fisher’s exact test. * *P <* 0.05, ** *P <* 0.01, *** *P <* 0.001, compared to WT. Data underlying this Figure can be found in S2 Data.
