# Supplementary material for: The Aedes aegypti siRNA pathway mediates broad-spectrum defense against human pathogenic viruses and modulates antibacterial and antifungal defenses
Source: PLoS Biol. 2022 Jun 9;20(6):e3001668. doi: 10.1371/journal.pbio.3001668 (PMC9182253; doi:10.1371/journal.pbio.3001668)
Supplement: S3 Fig — Log2 transformed fold-change is presented. Ae. aegypti Rps17 gene was used for the normalization. Data underlying this figure can be found in S2 Data. PBM, post-blood meal; qRT-PCR, quantitative real-time PCR; WT, wild type. (DOCX) [file pbio.3001668.s003.docx]

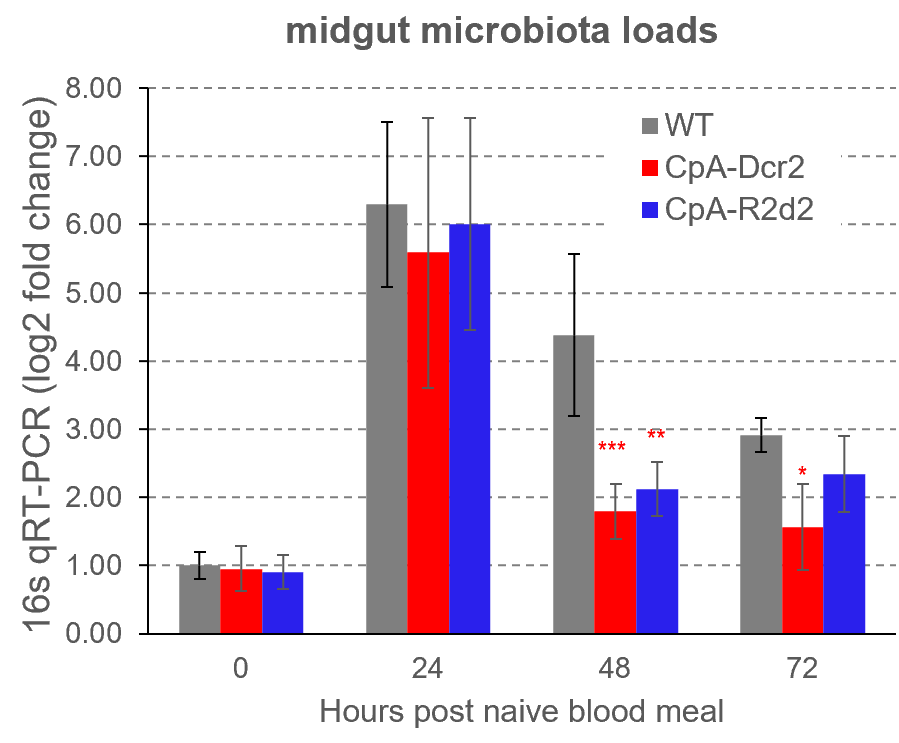


**S3 Fig.** The total bacterial loads of the midgut microbiota of female transgenic and WT control mosquitoes at 24-, 48-, and 72- h PBM (mean±SEM) were measured by qRT-PCR of *16s* bacterial ribosomal gene. Log2 transformed Fold-change is presented. *Ae. aegypti* *Rps*17 gene was used for the normalization. Data underlying this Figure can be found in S2 Data.
