# Supplementary material for: The Aedes aegypti siRNA pathway mediates broad-spectrum defense against human pathogenic viruses and modulates antibacterial and antifungal defenses
Source: PLoS Biol. 2022 Jun 9;20(6):e3001668. doi: 10.1371/journal.pbio.3001668 (PMC9182253; doi:10.1371/journal.pbio.3001668)
Supplement: S4 Fig — The survival rate of transgenic mosquitoes after oral challenge with either gram-positive (S. aureus: 800,000 CFU) or gram-negative (E. coli: 800,000 CFU) bacteria at 7 dpi (A, B). All groups of the mosquitoes in (B) were given a naïve BM to up-regulate the expression of the transgenes, while no BMs were given to mosquitoes in (A). The significance of the survival rates was determined by Kaplan–Meier survival analysis from 3 biological replicates (ns: not significant). Data underlying this figure can be found in S2 Data. BM, blood-meal; CFU, colony-forming unit; dpi, days post-infection. (DOCX) [file pbio.3001668.s004.docx]

**
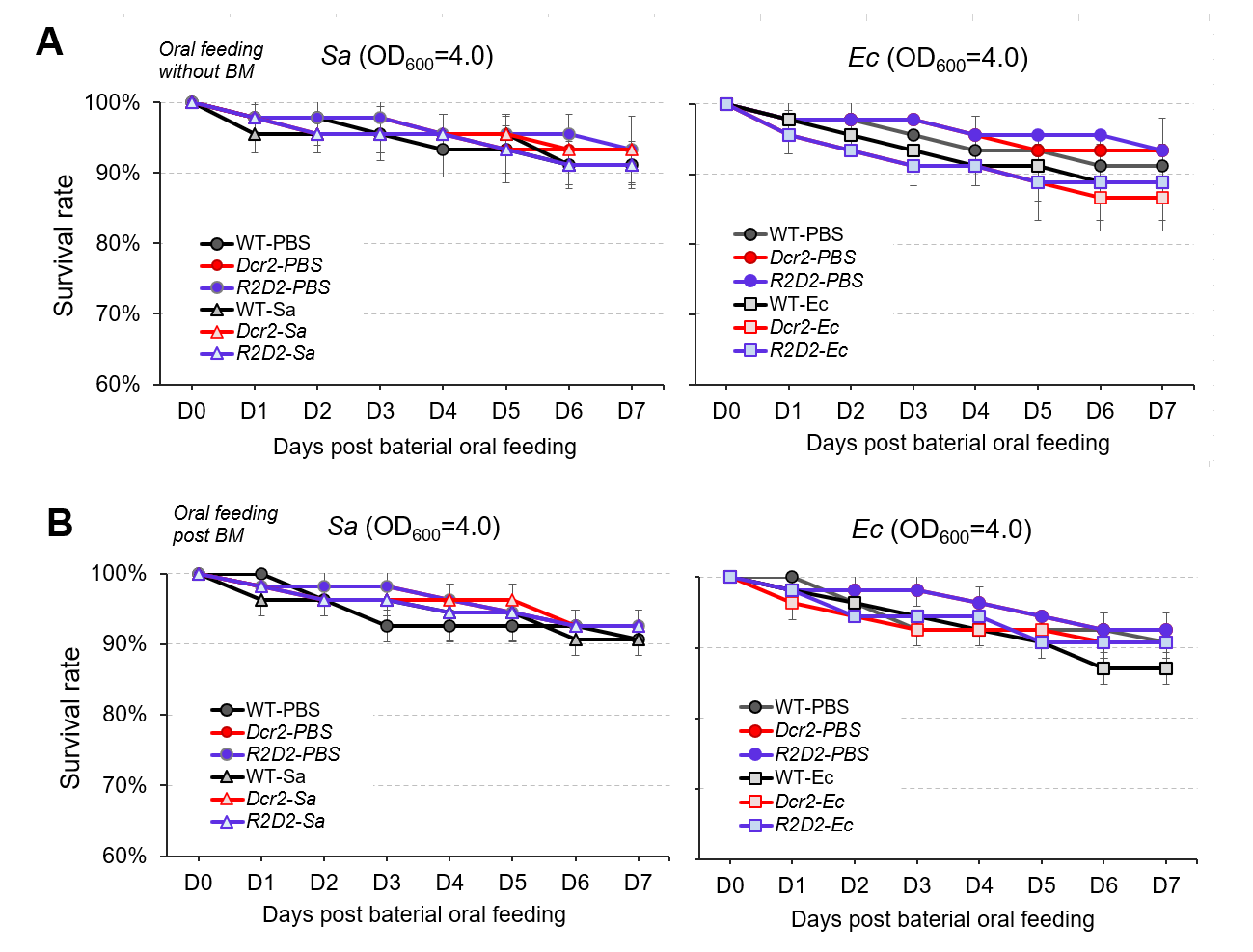
**

**S4 Fig.** The survival rate of transgenic mosquitoes after oral challenge with either Gram-positive (*S. aureus*: 800,000 CFU) or Gram-negative (*E. coli*: 800,000 CFU) bacteria at 7 dpi (**A, B**). All groups of the mosquitoes in (**B**) were given a naïve blood meal to upregulate the expression of the transgenes, while no blood meals were given to mosquitoes in (**A**). The significance of the survival rates was determined by Kaplan-Meier survival analysis from three biological replicates (ns: not significant). BM: blood-meal. Data underlying this Figure can be found in S2 Data.
