# Supplementary material for: The Aedes aegypti siRNA pathway mediates broad-spectrum defense against human pathogenic viruses and modulates antibacterial and antifungal defenses
Source: PLoS Biol. 2022 Jun 9;20(6):e3001668. doi: 10.1371/journal.pbio.3001668 (PMC9182253; doi:10.1371/journal.pbio.3001668)
Supplement: S5 Fig — The WT carcasses were used as the control, and the AeRps17 gene was used as an internal control for normalization. Data underlying this figure can be found in S2 Data. PBM, post-blood meal; qRT-PCR, quantitative real-time PCR; WT, wild type. (DOCX) [file pbio.3001668.s005.docx]

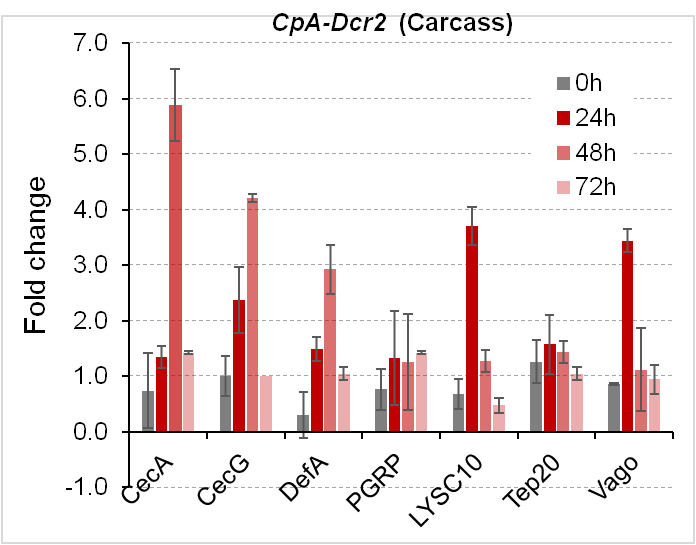


**S5 Fig.** qRT-PCR expression profiling of a panel of antimicrobial and immune genes in the WT and transgenic *CpA-Dcr2* mosquito carcasses at 0 h (before blood meal), 24 h, 48 h, and 72 h PBM. The WT carcasses were used as the control, and the *AeRps17* gene was used as an internal control for normalization. Data underlying this Figure can be found in S2 Data.
