# Supplementary material for: The Aedes aegypti siRNA pathway mediates broad-spectrum defense against human pathogenic viruses and modulates antibacterial and antifungal defenses
Source: PLoS Biol. 2022 Jun 9;20(6):e3001668. doi: 10.1371/journal.pbio.3001668 (PMC9182253; doi:10.1371/journal.pbio.3001668)
Supplement: S1 Table — (DOCX) [file pbio.3001668.s006.docx]

**S1 Table.** Primers were used to generate constructs for embryo microinjections to generate transgenic mosquitoes, verify transgenes on the transgenic mosquito chromosome, and qRT-PCR primers.

| **Names** | **Sequences (5' -3')** | **Notes** |
| --- | --- | --- |
| Dcr2F | ATGGATATGATTATGCCACAGC | The forward primer used for PCR of CDS or Dcr2 and ZeroBlunt cloning |
| Dcr2R | TTACTTAGCACTGCGGTAGTGC | The forward primer used for PCR of CDS or Dcr2 and ZeroBlunt cloning |
| R2d2F | ATGGCCTCAAAGCCAGTCCTG | CDS R2D2 amplification, Reverse primer used for ZeroBluct cloning |
| R2d2R | TCATCAATCAATGTAATCGAA | CDS R2D2 amplification, Forward primer used for ZeroBlunt cloning |
| Dcr2F_Avr _PBS | CTCCTAGGAAGATGGATATGATTATGCCACAGC | Amplification of Dicer gene for cloning into pBlueScript, Forward primer |
| Dcr2R_Pac_PBS | CTTTAATTAATTACTTAGCACTGCGGTAGTGCTTGCG | Amplification of Dicer gene for cloning into pBlueScript, Reverse primer |
| AeCpAF_Xho1_FseI_PBS | CCGCTCGAGGGCCGGCCCCAACGACAAAAGTCATGC | Amplification and cloning of AeCpA promoter into pBlueScript on the upstream of Dcr2 and R2d2 genes; Forward primer |
| AeCpAR_Nar_PBS | CCGGCGCCTTTCCCAACTAACCGACTGCACAC | Amplification and cloning of AeCpA promoter into pBlueScript on the upstream of Dcr2 and R2d2 genes; Reverse primer |
| AeCpAR_Hind_PBS | CCTAAGCTTTTTCCCAACTAACCGACTGCACAC | Amplification of the carboxypeptidase A promoter for cloning into pBlueScript upstream of the Dome gene, Forward primer |
| AeCpAR_Avr_PBS | AATCCTAGGTTTCCAACTAACCGACTGCACAC | Amplification of the carboxypeptidase A promoter for cloning into PBluescript upstream of the Dicer gene, Reverse primer |
| R2D2F_NarI_PBS | CTGGCGCCGAAAATGGCCTCAAAGCCAGTCCTG | Amplification and cloning of R2D2 gene into pBlueScript for generating CpA-R2D2-TrypT; Forward primer |
| R2D2R_XbaI_PBS | TTTTCTAGATCAATCAATGTAATCGAAATTAAGC | Amplification and cloning of R2D2 gene into pBlueScript for generating CpA-R2D2-TrypT; Reverse primer |
| pEntrCPA_F | CACCCGACAAAAGTCATGCGTG | Amplification of CpA-Dcr2-TrypT and CpA-R2D2-TrypT from pBluescript and cloning into pENTR/D-TOPO; Forward primer |
| pEntrTryp_R | GGTCGGCGCGCCCACCCT | Amplification of CpA-Dcr2-TrypT and CpA-R2D2-TrypT from pBluescript and cloning into pENTR/D-TOPO; Reverse primer |
| attR1 | CTAGGCCGGCCACAAGTTTGTACAAAAAAGC | Gateway cassette amplification primer for generating pMos1-attR |
| attR2 | CTAGGCCGGCCACCACTTTGTACAAGAAAGC | Gateway cassette amplification primer for generating pMos1-attR |
| CpA-GEO-VF | CGCGGTATAAGCTCCAATGAG | The forward primer of PCR verification primers for transgene CpA-Dcr2 and CpA-R2d2 |
| CpA-Dcr2-VR | CAAGTTGATGTGTTTAACCGA | Reverse primer of PCR verification primers for transgene CpA-Dcr2 |
| CpA-Dcr2-VR | CGGCACTCATATTCAAACTCT | Reverse primer of PCR verification primers for transgene CpA-R2d2 |
| 16S-qPCR-F | TCCTACGGGAGGCAGCAGT | qRT-PCR on bacterial loads; Forward primer |
| 16S-qPCR-R | GGACTACCAGGGTATCTAATCCTGTT | qRT-PCR on bacterial loads; Reverse primer |
| 16s27f | AGAGTTTGATCCTGGCTCAG | the forward primer of 16s of bacterial ribosomal gene for PCR and sequencing of bacterial isolates from mosquito midgut |
| 16s1522R | AAGGAGGTGATCCANCCRCA | Reverse primer 16s of bacterial ribosomal gene for PCR and sequencing of bacterial isolates from mosquito midgut |
| Rps17-F | CACTCCCAGGTCCGTGGTAT | ribosomal protein S17 gene for qRT-PCR normalization: Forward primer |
| Rps17-R | GGACACTTCCGGCACGTAGT | ribosomal protein S17 gene for qRT-PCR normalization; Reverse primer |
| AeDcr2-RT-F | CAACGCTTTCAGTCAAACGA | Forward primer for qRT-PCR of Dcr2 gene |
| AeDcr2-RT-R | ATTGATCCCCCAAAAAGACC | Reverse primer for qRT-PCR of Dcr2 gene |
| AeR2D2-RT | GACCTACCGGGAACTCATCA | Forward primer for qRT-PCR of R2d2 gene |
| AeR2D2-RT | GATCAGGGTGCATTTGTCCT | Reverse primer for qRT-PCR of R2d2 gene |
| RTAaAgo2_F | CGAGATGATTAGAGATCTGC | Forward primer for qRT-PCR of Ago2 gene |
| RTAaAgo2_R | ATGGCACGAAGTTCTATGG | Reverse primer for qRT-PCR of Ago2 gene |
| RTAeVag_F | CTGTCGATAGTGGCTGACCC | Forward primer for qRT-PCR of Vago gene |
| RTAeVag_R | TGATAACTCACTTTTCCATCGTGT | Reverse primer for qRT-PCR of Vago gene |
| MLF1 | TTGTTTACTCTCAGTGCAGTCAACATGTCG | Inverse PCR of pMos left arm forward primer |
| MLR1 | TTCGACAGTCAAGGTTGACACTTCACAAGG | Inverse PCR of pMos left arm reverse primer |
| MRF1 | AAGACGATGAGTTCTACTGGCGTGGAATCC | Inverse PCR of pMos right arm forward primer |
| MRR1 | CTTGCCGTATGTGATGGAGCGTTGTCATGG | Inverse PCR of pMos right arm reverse primer |
| AeCECG-qPCR-F | GTTATTTCTCCTGATCGCCG | Forward primer for qRT-PCR of CecG gene |
| AeCECG-qPCR-R | CTCGTTTTCCTGCACCTCCC | Reverse primer for qRT-PCR of CecG gene |
| AeCecA-F-qPCR | CAAAGTTATTTCTCCTGATCG | Forward primer for qRT-PCR of CecA gene |
| AeCecA-R-qPCR | CTGCACCTTCCAATTTCTTTCC | Reverse primer for qRT-PCR of CecA gene |
| AeDEFA-qPCR-F | CTATCAGGCCGCCGTGGAGAA | Forward primer for qRT-PCR of DefA gene |
| AeDERA-qPCR-R | CAATGAGCAGCACAAGCACTATC | Reverse primer for qRT-PCR of DefA gene |
| AeTEP20-qPCR-F | TCTCTTACCTTGGAGGCCTATT | Forward primer for qRT-PCR of Tep20 gene |
| AeTEP20-qPCR-R | GTCAGGCTTTCTCTTGGTATGG | Reverse primer for qRT-PCR of Tep20 gene |
| AeLYSC10-qPCR-F | GACACCAACAAGAAGCACAAC | Forward primer for qRT-PCR of Lysozyme P gene |
| AeLYSC10-qPCR-R | CTGTGGATCGCACCAGTATC | Reverse primer for qRT-PCR of Lysozyme P gene |
| AeVago-qPCR-F | GACCTTTACGAGCTGTGTACTG | Forward primer for qRT-PCR of Vago gene (new primer) |
| AeVago-qPCR-R | GTGACAACACTCCGGGTAATC | Reverse primer for qRT-PCR of Vago gene (new primer) |
| AePGRPLB-qPCR-F | TCTACGAGGGACGAGGATTT | Forward primer for qRT-PCR of PGRP-LB gene |
| AePGRPLB-qPCR-R | CGTCCAATCTCCGATCATACAA | Reverse primer for qRT-PCR of PGRP-LB gene |
| RT rpS7 F | GCAGACCACCATTGAACACA | Forward primer for qRT-PCR of rpsS7 gene for normalization |
| RT rpS7 R | CACGTCCGGTCAGCTTCTTG | Reverse primer for qRT-PCR of rpsS7 gene for normalization |
| ZIKV-forward | AGCAACATGGCGGAGGTAAG | Forward primer for qRT-PCR of ZIKV viral RNA copies |
| ZIKV-reverse | CTGTCCACTAACGTTCTTTTGCAGA | Reverse primer for qRT-PCR of ZIKV viral RNA copies |
