# Supplementary material for: The Aedes aegypti siRNA pathway mediates broad-spectrum defense against human pathogenic viruses and modulates antibacterial and antifungal defenses
Source: PLoS Biol. 2022 Jun 9;20(6):e3001668. doi: 10.1371/journal.pbio.3001668 (PMC9182253; doi:10.1371/journal.pbio.3001668)

**S2 Table.** DNA sequence obtained by inverse PCR flanking the transposon Mos1 random insertions from both *CpA-Dcr2* and *CpA-R2d2* transgenic constructs. Inverse PCR was done using primers (MLF1 and MLR1) in S1 Table**.**


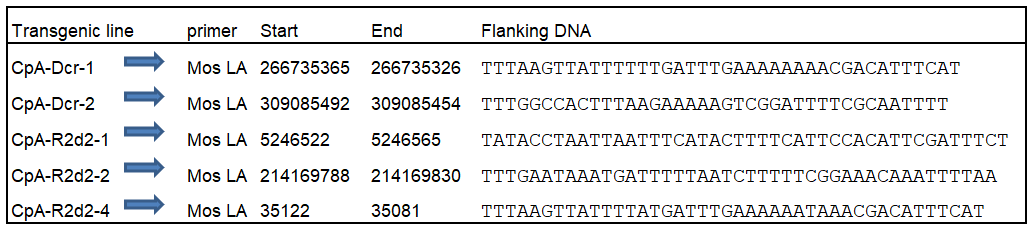

Supplement: S2 Table — Inverse PCR was done using primers (MLF1 and MLR1) in S1 Table. (DOCX) [file pbio.3001668.s007.docx]
