# Supplementary material for: The Aedes aegypti siRNA pathway mediates broad-spectrum defense against human pathogenic viruses and modulates antibacterial and antifungal defenses
Source: PLoS Biol. 2022 Jun 9;20(6):e3001668. doi: 10.1371/journal.pbio.3001668 (PMC9182253; doi:10.1371/journal.pbio.3001668)
Supplement: S3 Table — (DOCX) [file pbio.3001668.s008.docx]

**S3 Table.** Enriched immune genes related to the immune pathways in the *CpA-Dcr2* and *CpA-R2d2* transgenic mosquitoes at 24 h post blood meal (detailed gene list from Fig. 3, (**h** and **i**)).

Statistical significance levels: a, *P* < 0.05; b, *P* < 0.01; c, *P* < 0.001; d, *P* <0.0001.
