# Supplementary material for: The Aedes aegypti siRNA pathway mediates broad-spectrum defense against human pathogenic viruses and modulates antibacterial and antifungal defenses
Source: PLoS Biol. 2022 Jun 9;20(6):e3001668. doi: 10.1371/journal.pbio.3001668 (PMC9182253; doi:10.1371/journal.pbio.3001668)
Supplement: S1 Data — (DOCX) [file pbio.3001668.s009.docx]

**S1 Data.** The maps and the sequences of the plasmids were used for embryo microinjections to generate *CpA-Dcr2* and *CpA-R2D2* transgenic mosquitoes.

**> *pMos1-AeCpA-Dcr2-3xP3-dsRed***

GCCTCGTGATACGCCTATTTTTATAGGTTAATGTCATGATAATAATGGTTTCTTAGACGTCAGGTGGCACTTTTCGGGGAAATGTGCGCGGAACCCCTATTTGTTTATTTTTCTAAATACATTCAAATATGTATCCGCTCATGAGACAATAACCCTGATAAATGCTTCAATAATATTGAAAAAGGAAGAGTATGAGTATTCAACATTTCCGTGTCGCCCTTATTCCCTTTTTTGCGGCATTTTGCCTTCCTGTTTTTGCTCACCCAGAAACGCTGGTGAAAGTAAAAGATGCTGAAGATCAGTTGGGTGCACGAGTGGGTTACATCGAACTGGATCTCAACAGCGGTAAGATCCTTGAGAGTTTTCGCCCCGAAGAACGTTTTCCAATGATGAGCACTTTTAAAGTTCTGCTATGTGGCGCGGTATTATCCCGTATTGACGCCGGGCAAGAGCAACTCGGTCGCCGCATACACTATTCTCAGAATGACTTGGTTGAGTACTCACCAGTCACAGAAAAGCATCTTACGGATGGCATGACAGTAAGAGAATTATGCAGTGCTGCCATAACCATGAGTGATAACACTGCGGCCAACTTACTTCTGACAACGATCGGAGGACCGAAGGAGCTAACCGCTTTTTTGCACAACATGGGGGATCATGTAACTCGCCTTGATCGTTGGGAACCGGAGCTGAATGAAGCCATACCAAACGACGAGCGTGACACCACGATGCCTGTAGCAATGGCAACAACGTTGCGCAAACTATTAACTGGCGAACTACTTACTCTAGCTTCCCGGCAACAATTAATAGACTGGATGGAGGCGGATAAAGTTGCAGGACCACTTCTGCGCTCGGCCCTTCCGGCTGGCTGGTTTATTGCTGATAAATCTGGAGCCGGTGAGCGTGGGTCTCGCGGTATCATTGCAGCACTGGGGCCAGATGGTAAGCCCTCCCGTATCGTAGTTATCTACACGACGGGGAGTCAGGCAACTATGGATGAACGAAATAGACAGATCGCTGAGATAGGTGCCTCACTGATTAAGCATTGGTAACTGTCAGACCAAGTTTACTCATATATACTTTAGATTGATTTAAAACTTCATTTTTAATTTAAAAGGATCTAGGTGAAGATCCTTTTTGATAATCTCATGACCAAAATCCCTTAACGTGAGTTTTCGTTCCACTGAGCGTCAGACCCCGTAGAAAAGATCAAAGGATCTTCTTGAGATCCTTTTTTTCTGCGCGTAATCTGCTGCTTGCAAACAAAAAAACCACCGCTACCAGCGGTGGTTTGTTTGCCGGATCAAGAGCTACCAACTCTTTTTCCGAAGGTAACTGGCTTCAGCAGAGCGCAGATACCAAATACTGTCCTTCTAGTGTAGCCGTAGTTAGGCCACCACTTCAAGAACTCTGTAGCACCGCCTACATACCTCGCTCTGCTAATCCTGTTACCAGTGGCTGCTGCCAGTGGCGATAAGTCGTGTCTTACCGGGTTGGACTCAAGACGATAGTTACCGGATAAGGCGCAGCGGTCGGGCTGAACGGGGGGTTCGTGCACACAGCCCAGCTTGGAGCGAACGACCTACACCGAACTGAGATACCTACAGCGTGAGCTATGAGAAAGCGCCACGCTTCCCGAAGGGAGAAAGGCGGACAGGTATCCGGTAAGCGGCAGGGTCGGAACAGGAGAGCGCACGAGGGAGCTTCCAGGGGGAAACGCCTGGTATCTTTATAGTCCTGTCGGGTTTCGCCACCTCTGACTTGAGCGTCGATTTTTGTGATGCTCGTCAGGGGGGCGGAGCCTATGGAAAAACGCCAGCAACGCGGCCTTTTTACGGTTCCTGGCCTTTTGCTGGCCTTTTGCTCACATGTTCTTTCCTGCGTTATCCCCTGATTCTGTGGATAACCGTATTACCGCCTTTGAGTGAGCTGATACCGCTCGCCGCAGCCGAACGACCGAGCGCAGCGAGTCAGTGAGCGAGGAAGCGGAAGAGCGCCCAATACGCAAACCGCCTCTCCCCGCGCGTTGGCCGATTCATTAATGCAGCTGGCACGACAGGTTTCCCGACTGGAAAGCGGGCAGTGAGCGCAACGCAATTAATGTGAGTTAGCTCACTCATTAGGCACCCCAGGCTTTACACTTTATGCTTCCGGCTCGTATGTTGTGTGGAATTGTGAGCGGATAACAATTTCACACAGGAAACAGCTATGACCATGATTACGCCAAGCTCGGAATTAACCCTCACTAAAGGGAACAAAAGCTTTATGGTTTATGATTATCAATTTCGTTCCCATTGCTGGAATATCTTTCGGGTTGTTTGTTATGCCTCGCTTTTCACTGCGTGCTAGCAGTTCCCTTTTCCTTAGGAATGACCATGCGCACTTCCACTTTCGTGGGATAAAAATGGTAAATCCGGCTTTTTCTTAAAGCTACCAAATGATGTAGATAGGCATCCCACAGTACGGGCTCCAGTCGGTGAATATAGAAACTATCAGGTGTACAAGTATGAAATGTCGTTTTTTTTAAATCAAAAAACACGTTAAATTTTGTGGAAAAAGAAAAAATCATTTATTCAAAGTATTTGCCGTCGCTAGCTACACATTTTTCCCATCTCTCGGGCAATTTGTGGATTCCACGCCAGTAGAACTCATCGTCTTTTGCGGCGAACCATTCATCGAGCCATTTTTTCACACTTTCGTAAGAATCGAAGCGCTGCTCAGCGAGTGCGTGTCCCATCGAAGCGAATAGGTGGTAATCGGATGGGGCCAGGTCTGGTGAGTAAGCCGCATGCGGAAGCACTTCCCAATTGAGTGTTTCCAACGTGTCGCGAACCGCTCTTGCCGTATGTGATGGAGCGTTGTCATGGAGAAAAATGACCCTGTGTTGTCTTTTTTGATATTCCGGTCGTTTTCTCTGAAGCGCACGGTTCAAATTGATCAATTGTTGTTGGTAGCGTGCCGTATTCACCGTTTCGCCGGGTTTCAAGAGCTCATAGTAAATGACACCGCTCTGATCCCACCAAACACAGAGCATCGTCTTCTTGCCAAAGCGATTCGGTCGAGCAGTCGATGTGGCCGGTTGTCCAGGATCAACGTATGACTTTTTACGTTTAGGATTAACAAAAAAGATCCATTTTTCATCTCCAGTAACGATACGATGCAAAAACGACTTCCTTTTGTATCGTGAAAGCAAAATTTCGCATGTGTTTTTGCGCCTCTCCATCTGCCTCTCGTTCAACTCATGTGGCACCCATCTACCGACCTTCTGAATCTTTCCCATCTCTCGCAAGCGATTGGAAACTGCTTGTTGACTTACTTCCAACTGCTCTGCGAGTTGTTTTTGCGTTTGAGCATCGTCTTCATCCAATAATGCTTGCAGTTCGGCGTCTTCGTACCTTTTTGGCGGTTTTCCGTGCTCTTTGTCGTCGAAATTCGAGCTCGCCCGGGGATCTAATTCAATTAGAGACTAATTCAATTAGAGCTAATTCAATTAGGATCCAAGCTTATCGATTTCGAACCCTCGACCGCCGGAGTATAAATAGAGGCGCTTCGTCTACGGAGCGACAATTCAATTCAAACAAGCAAAGTGAACACGTCGCTAAGCGAAAGCTAAGCAAATAAACAAGCGCAGCTGAACAAGCTAAACAATCGGGGTACCGCTAGAGTCGACGGTACCGCGGGCCCGGGATCCACCGGTCGCCACCATGGCCTCCTCCGAGAACGTCATCACCGAGTTCATGCGCTTCAAGGTGCGCATGGAGGGCACCGTGAACGGCCACGAGTTCGAGATCGAGGGCGAGGGCGAGGGCCGCCCCTACGAGGGCCACAACACCGTGAAGCTGAAGGTGACCAAGGGCGGCCCCCTGCCCTTCGCCTGGGACATCCTGTCCCCCCAGTTCCAGTACGGCTCCAAGGTGTACGTGAAGCACCCCGCCGACATCCCCGACTACAAGAAGCTGTCCTTCCCCGAGGGCTTCAAGTGGGAGCGCGTGATGAACTTCGAGGACGGCGGCGTGGCGACCGTGACCCAGGACTCCTCCCTGCAGGACGGCTGCTTCATCTACAAGGTGAAGTTCATCGGCGTGAACTTCCCCTCCGACGGCCCCGTGATGCAGAAGAAGACCATGGGCTGGGAGGCCTCCACCGAGCGCCTGTACCCCCGCGACGGCGTGCTGAAGGGCGAGACCCACAAGGCCCTGAAGCTGAAGGACGGCGGCCACTACCTGGTGGAGTTCAAGTCCATCTACATGGCCAAGAAGCCCGTGCAGCTGCCCGGCTACTACTACGTGGACGCCAAGCTGGACATCACCTCCCACAACGAGGACTACACCATCGTGGAGCAGTACGAGCGCACCGAGGGCCGCCACCACCTGTTCCTGTAGCGGCCGCGACTCTAGATCATAATCAGCCATACCACATTTGTAGAGGTTTTACTTGCTTTAAAAAACCTCCCACACCTCCCCCTGAACCTGAAACATAAAATGAATGCAATTGTTGTTGTTAACTTGTTTATTGCAGCTTATAATGGTTACAAATAAAGCAATAGCATCACAAATTTCACAAATAAAGCATTTTTTTCACTGCATTCTAGTTGTGGTTTGTCCAAACTCATCAATGTATCTTAAAGCTTATCGATACGCGTACGGCGCGCCTAGGCCGGCCACAAGTTTGTACAAAAAAGCAGGCTCCGCGGCCGCCCCCTTCACCCGACAAAAGTCATGCGTGTTAATTGAATGGTGTGGAGCAGGAGCGGGTACAAGGGATAATATTGCGGTGCTCATTTGAAATTCACATCCAGCGGCGGCAGTAACGCGCAATTCAAACGCAACTCTGAAATTCAATTAATCCGATAATCGCGTTCCATGGATTTTTCTTGCAGAGCACAATATGATGAATCAATCAACAGAAATGTCACGCTGCAACTTCTTGGTTGGTTGATTGGTTCTTGGTTGGATTCACTCCTGCTGCAAGTTTATAACCATGACATGGCAATTTTTACAGCCAAAATGCTGCAATAGTGAGATTTTATACAAAAAATATTCGATGAAATAAATGAAAACGAAATTAGGCAGCGTCCATTTATGACGTAAAGCTAAAATGTACAATTTTCGACCTCCTCCCTCTCCCGAAAGACCGTTGTTCTCAGTGTAGTCTGTATTTTTCTGCATTGGCCCTTTCAACAAGTAGAACATAATGAGCGACGTTTAATTTCGGGATTTGTCAACAAATACGAAATGTTACCAATACATAAACTAGTTTTTGCACAGTTTTGGATTGCCGAAGAGAACATTTTAGTTGCCGACAATAGTAATTGCATTGCCGATAAAAGAACAAGTGCCTCGTGACTTTCTTTAGGAAAAATAGATGATTTAGAGAACAAAATAGTGTTTTGTGTATAATAACCAATAAATGAAGACTGCTCAAGTGTAGTTCAGGTGTCTCCTGCTAATTGTTGTGATTTATTGTTGTGTATAACTGCCTTTTTAAAAGTTCAGCTGTTTAGAGAATACGTAAATTCCCCTATATTTTTCACTGCCTGCGAGGTGATTTTGTTTTGTACAACGCCACTAATGAGTTTTAATCACGTTTTGTTCTGAAAGATGACCCTTAGTTGATCCATAACTGTGGGGTGACTGTACTTTACACAACGGAGAGATCTGTGACTGACCAACGTGACATGGAATTCGTCAATAAAAAAATTCGTTCAAAGCAAGATATTTTAAGAGTGTTTAAGCATTTATTTGTTTTTGAATTGTAATGGGTTGAGTTTTAAAAGTCTTGTTGAGCTAGTGAACCAAAGAAGCATTTAAAACTAGTTTAAAAAAGTTTAAAACTACGTTTTGTAATGAGAACTTTAATATCTCAATTCAAAACCAGAAAGTTGATACGTTTCTTTTATCTAATGCTTAGCCACCTTAGTTATCTTTTATCTAATGCTACAACATCACCAAATATTACTAAAAAAAGCCCTTCTAACTGTAGACTCAGAGTCCCGAATTTGTAAATTCTGCCCTGTTTTCCATGGTAGATTGTTTAAATTAGAATACCTGCTGTGAACCTATCCGACTTCACCCTACTGTTCTTTAAGGCCACCATGCTTTGAATATCGCTTATTTTAAAAGTCAAAGCGATTCCTATCATAATCAGTGGAAAAAACGTTAAATAAAACTAAGAATGAATCCGTCAGCATGGATCAAAGGCAGACTTCTACTTTCGAGTACCCAACGGGAGTTAAATCGCGGTATAAGCTCCAATGAGGTACCGTAGCTGATAAGACAACTATTTCCACTTAACGATGTGCTCAAGCGAACTATAAATGGCACCTGATTGCAATTGGCCAGGTTAGTGTGCAGTCGGTTAGTTGGAAACCTAGGAAGATGGATATGATTATGCCACAGCAAGACGATTTCATCCCGCGGGACTACCAGCGGACGATGAAGACAATCTGCATGCAGAAAAATACAATCATCTACTTGCCGACCGGAGCCGGAAAGACCCATATCGCCCTGATGGTCATCAAGGAAATGGGCAAAGATCTGGATAAGCCATTAACTGGAGGTGGTAAAAGGACGTTTTTTGTCGTGAATACCGTTGCATTAGCTAAGCAGCAGGCCGAATTCCTGAGTCATAATCTTACCTACGACACATCGATCTATACCAGTGATCGAAATGTGGATGCTTGGAAACAGGACAAATGGCTGGAAGAGTTCGCGAAGTACCAGGTCATTGTTTGCACATGTCAAATATTGTTGGACGTGCTGAAGCATGGTTATCTCTCGGTTAAACACATCAACTTGCTTATATTTGATGAATGCCACCATGGTGTTGGAGAACATCCTATGCACGGGATTATGGAACAATTTCTGAGGGTTCCAAAGTCTGACCACCCACGTGTAATCGGTCTTTCTGGAATGTTACTCTATAAACAGATTAAAAGTGTCGCCCTAGTATCTCCAGAATTGGAACGCTTGGAAAATACATTTAACGCGACAATTGCTACCGTTGGGAGTTATGATGCTTTCACCGAGGTCTGCAAATTTTCGACAGATCCCAATGAACTTTTGGTGTCTTATTCAACCCTTCGACTGTCACCTGTGATGGCTGACATCGTGAACAACATCAACGCTTTCAGTCAAACGATTGAAGAATTTCACCTTCCAAAATATTTAAATCAAAATAAAGCCCTCCTAAAAGACAGACCAAAGCCACTGAAAGAGATCCGAAAACTCTTCACAGAGTTGATTTATCAGCTTGGCGATACTGGTCTTTTTGGGGGATCAATAGCTCTACTTGGTTTGATAGTCCAATTTGAGCTGGACAAAAGGCAATCGGATAGTTCAATGCTAAGACTGGCGTTGAGGTCGTGTATTACTTTTTGCGAAAGCCTGAGGCATCAGATCGAAAAATTAATGAGTGGTTTGGACATGAAGACCAAATTGACTAAGTTCAGCTCTCTGAAGGTCCGCCAATTGATAGATCAGCTGGAAAAGTTGTACGAAGAGAATCGTGACAAAAAAGCGAAAACGCTGATTTTCGTCCAGCGACGGTTCTCGGCGAAAGTTTTGTACCATTTACTGAAAATTTACTTCGCTGAGACGGAAGACGCCAACCTTATTGTTCCAGATTTCATGGTGGGCAACAATGGTTCCATGCCCGAATCGATTGGGCAAATTTTGAGTGCTAAAAAGGATAGAAGGGTTCTCGAACGGTTTAAAAAGAATGAAACAAACGTTATTGTAACAACCAACGTGTTAGAGGAGGGGATTGATCTTCAAATGTGCAACACTGTCGTCAAGTATGACCATCCGCAAACATTTGCGTCGTATCAGCAGTCAAAAGGAAGAGCCCGTATGAAGAACAGTCAGTATATGGTAATGCTGGATAACGAAAATCGTCATATCTTCCTGGAGAAATACAGACTCTATAAGAGTATTGAAGAGGAGTTGCGAAGGTGCCTTATCGGCAAAACAATAAACCGGCCAGACCCCCTCGATGCGGATGTCCACAAAGAGCTATACAATGAGATCATTCCACCGTTTTTCACCGCCAAGGGTGCCAAGCTGGATGCTCTGTCGGCCATACAGCTCCTGAACCGCTGCTGCATGGGAATGCCGAGAGATGCATTTACCAATACTAACGTCACTTGGGAACGGATCGATCTGAAGGACGGCAGAATAATAGTGGAAGTTTTGTTGCCGCTTCAGTCCACCGTGCGAGAAAAAATCTCCGGCAATCCCATGCGCAATATCAAGTTGGCAAAACGATCAGCGGCGTTCAACGCGTGCCGAAAACTGTATGAGAATAAGGAGCTGAATGAACATCTAATACCCATAGACTGCAAATACCAGCTTAACAATTTGAAAGATGTGTATTTCCGTCATTGGAAGGATTTTGATGCAGACCTCGGCAAACTAGCAGGCACCCAAAAGTGCATACGAACGCACGCTATCCAGTATCCAAAGCAAACAACAGAGTGTTTCCCACAGCCGGGCAAACCCTGTTACATCTACGTTCTGCGAATTGCAGCTGGGTTTGCACAGGATCCAACAAACGATAATGTCAACATTTTCCACTCTTTGTATAGCTCGGAGAATAATTTTGGATTAGTGACTACGAAACCTCTCCCGGCCCTAGCAAAGATGAAGTTCTTTGTGACTTTGGGACTAATCAACGTACACATAGAGGAGACTCCCATCGCGCTGCCTAACGGGGGATCCGAAATAGAGCTTGCTCTACTGAGACAATTCCATGTTACTGTCTTCCGCGACGTGCTAAAACTGTGGAAGGAATTTCTCTGCTGCGATTACGACAACGAGGAAAACAGTTTTCTGGTGGTGCCTTTGAAAAATTCTACTCACCTAGACTGGAAACTCATCCGAGAATTCCAAAATTTGAGCGAACCACCTTCGGAAATTTCAACGATAGCTCGTAACAAGATGGAATTCGAAGCCGATAAGTACCGACACAAGGTCATTTTACCGTGGTACAAGAACAACAAGGAACAGCCATACGTTGTCACTATGGTGCATGAACACCTGACTCCGGAGAGTCCTTTCCCAAATCCGGAGTACGGTTCCTACGCGAATTATTTCAGCCAAGCTTACCATTTGGCAGTGGTTAAGCCAGATCAGTTTCTCATTGAAGTGAAAGGCATCACTAGTTACCTCAACCGGTTGAACCCCGGAGTGGAAGACGACGGAAAGAGCACCCGAAGCAAACATTGGCGTTTTAACGAAATTCTGATTCCCGAACTGTGTCACAACTACCAATTTCCAGCTGACTACTGGTTGAAGGCCACCCTCCTGCCCAGTGCACTTCATCGATTACACTATCTTCTGTTGGCGGAAAACATTCGCGTGGATCTGGCAACGGGTGCAAATGTTGGCTGTTTGGAGAATCACACGATCGAAGACGTGGACGTTGAGTACAAGGAGCGGAAAGGAAAGCAGTTGGAGGAACTACAGCTGATGGAATTTGAAGAAGATGAAGACGAAGACGACGAATTTGATTTGGAAGGAGCGAAGAGGTCATTGGTGGCTCCAGAGAATTTGAGCGAGTTGGCACGAAACCAAATGTGCTCCATTACCGGCGATATACCTCTGCCGTGGCAGGAGGACGAGGAACCGGTGGATATTGAGCGAAACTGGGATCAAGTGTCGAAACTTGATCTGGACTACTACAATGTCTTTGTGAATAAGTTTTCCGATCTGTCGATGCGTGAAAAGGCCGCAGAGCGTATTAGTAATGCATACACGTCAGCCGTATATAGACGTGCTGCTGGTAGTCCGAAGCGAGAAGCTATGGCAATCTTGGATGTACCCGTAGACCAGAAATTTGCCATTAAACTGCTCCAGTTGACCCCAGCAAATACGGTCAATGTCAATCTTCAACAGAAGAACATAATCAAGGCATTGACGACGAAATCATCGTCCGATGTTTTTGACCTGGAACGCTACGAATTGTTGGGCGATGCTTTTTTGAAGTTTTCCATCTCTCTCTACCTTGTTAAATATCACAAGGAATGGCACGAAGGCTTCCTCACTGCAGTTAAGGGCCAAATTGTAAGCAATCGCAACCTGGTCTATTGTGCGATAAAGTACGGACTACCTGGAATGCTAAAAATTCACAAATTCGATCCCAAAAACGACTGGCAACCACCTCTAGCAACGGTTCCGAAGAACATCAAACGAACGATGCAATCTGTGAATCATTCCGCCCGAGTGCTATATCGGTTGACCTTAACAGAGGAGGAAATCAAAACCGGTGTGGTGACAGCAAAGAACAGTGATGATTTTATTGCTCAGCTTGAGCTGCATGGCAACATGCCTGATCCCTCGCCAATGGCAAATTATCTCTCACAGCAGACCATGGGGGACAAGACACCGGCGGATGCCATGGAAGCATTGCTAGGCGTCTGTGTGCAATCAGTTGGCATCGAGCGTTCCTTCAAACTGTTGCCACATTTCGGAATCCTGCCGAAAACGCACAATGTCCTGAAGCTCCTTGCTGACAAGATCGAAAACCAACGACTGAAAACTCATATTGATATTCGTGAAGTAGATGCATTCCTCAAGAACTACAGAAGAATTGAAAGTATTCTGGGATATAAGTTCAAGGATAGAACTTATCTCCTACAAGCACTCACCCATGCGTCATACCCTACCAACAGAATTACGGGAAGCTACCAACAGTTGGAATTCCTCGGCGATGCGGTATTAGATTTCCTCATCTCGATGTACATTTTTGAGCAGAACCCTACCATGAGTCCCGGGCAGTTAACAGATCTGCGCTCGGCTTTGGTGAATAACGTCACTTTGGCGTGCATACTTGTTCGCCATGGTCTCCACTTGTACATCCTGGCAGAGTCGGCATCTTTCACCGACACAGTTAGCAAGTTTGTGTTGTTCCAAGAACAAAACAAACACGAAATTACCGATCAGGTGAACTTGCTCGTCGAAGAATCAGACCGGAAGATGGCCGAATTCGTAGACGTTCCGAAGGCGCTGGGCGATGTGTTCGAGAGTCTGGTAGCTGCGGTCTTTTTGGACTCTGGAAACGACTTTGCCGCCACCTGGCAAGTCATTTACGGCATGATGGGCAATGAAATATTGACCTTCACCGAGAACACACCGATCCAGATCGTGCGACAGTTGTATGAGTTCAAGCCGTCGTGCAAGCCAACCTTCAGCAGGGCCATCCCGGACGAGGACACGGTGCTTGTTAAGCTGCGGTACGAGATAAGGAACCAACAACACGAAGCGTATGGTTTTGGTCAAAACAAGGACGACGCAAAGAGGGCAGCGGCCAAAGCGGCACTGCAAGTGTTGCACAAGCACTACCGCAGTGCTAAGTAATTAATTAATAGGTAGCTGAGCGCATGCGATCTCGGCTTCAAAACGGTACTGGATTTTGGATTCAAACGAAAGCCATCGCTACAACAGAACAAATAAAAGAACATTAATCAAAACGCATAAAAGATGGGTTAATTGTATTCAATAAGGAGAAAAGTAATTCCTACTAGATAGTTTACTATCACGCGAAAGGATGGCCAGTCTTCACTACGGGAAGACAACCTCGCTGGGAATCGAAACTCTGTCAACGCTGGAGGTGCCAACACATCTTCGTAATAAACATTTTTACATTTATCCAGGCGTAAAGAAACACGATTTAGTTATCAATTTGTATTTTTGGTTCTTATGAAGAATAAACTTCTTCAAATTCACTCCCACGAATATTCCGTCCCGTTCCGCCAGTTCCATTCGAGCTCAAGGGTGGGCGCGCCGACCAAGGGTGGGCGCGCCGACCCAGCTTTCTTGTACAAAGTGGTGGCCGGCCGATCTCGTCGACGTCAAAATCACCACTTTTGAAGCGTTGAAACCACCGTTCACACGTTTTCACAGTTGGTACTTGTTCGCCAAAGGCTTCAACAAGCATTCGGTGCGATTCCGCAGCTGTTTTCTTCAAATGAAAACAGAAAATTAATACTGTCCGCGTTTGCTCTTTATTCGGCACGAAACTCGACATGTTGACTGCNCTGAGAGTAAACAATTATGACGCTCAATTCGCGCCAAACTATGGTGGTTCGACAGTCAAGGTTGACACTTCACAAGGTCAAAGTTTTATGACAATCGATAAATATTTACGTTTGCGAGACATCTATATGTTCGAACCGACATTCCCTACTTGTACACCTGGTAAAAAATTAAAACTAGGTCCCCGTTCTTACGCATATATACACAGGTACGTGGGTGTTAATTAATTACTTTAAAACATTAAGGAACTTCGATTGGCATTTAACAAGGAAACTAACATCATATTGTTATACAAATAAAAGTTGCTCATTTCTTACTCGTAATTCGAAACCTTTCTCGGTTCTTTTTTTTACCATACTATCACTTTAAAGTGGTTTTAACCAACATGCATACATTTCGTTTTTTTTTCGTTTTACTCGTCTGCGTTCGCTCTTTGTTTTTTAACCAAAGTAAATCTGACTCAGGGAAGTCACTCAAAACATGTTTGCCTGAGCTCTCGTATTCCAAATGGAACTCCGGGAAAAATATATGCTCAAAAGTGGCTCAAAACTGTATCTATTCGTATTTTGATATACACAATAATAAAACTGCTCGAAGAACTCCAATCTGTTACACAATGCCTGGTCATTGTTTACATTTAGCTCACTTGTGTACTTATGTGCACCCATACACACCCATGTGTAGATAAAGGATGGCTTATCTTATCACTGACAAAACAGCGGAGCATGCAAGAATGCTGTTTGTCTTTGTCCATTGGCTTCGATTAAGTAGTCGAAAAGGAAAACGGTAAATGGAATGCATAACTTTGCCCTCTCACTCCCTCTAATCGGACGGAAGAGTCGGACGTTATGACCTGTCCTGATTTGGAGAAATCTTTTCTATATTTCATTTATTTCGCTTACTTCCCTACTTCTTTCAAATGCACTAATGCGAAAAACATTATTTTTTTCAAATTAAATGGTCAAGTGTGCTTCTAATGAAATCCATAGTCTCAATACATACATATATGCAGTCGGCAAAAAAGGAGGCACATACTCGTAGAAATTTTTCATTGCTCTGTATATGGATTTGTAAATGGTATAAAAAGCATATATCAGTATCAGTTCTCTTCCCATTCCCGCTCTCAGTGCGCTCTCTCGGTTGAGCAGTTTTCGAGCCTCTCGAGTTCGAGTTCACCGAGTTCGAGTTGCGACGGTTCGCCCACTCAGTTTGCCCACAGCTCTGAATGCGAGCGTAACGATCGCAAGCCGCCTGTCTTGCAGTGAAGTGCATTTTCTCAAGCCGCTTCCTCGATTTGTATTTGGTTGGACAACAGCCCATAACTTGAACCCGTCTTTAATGCCCGTATCTCTGCGAGAGTGAGTTTCCAATGGCGATTTGCCGAGTGCGCGTGTGCAGGGCACCCTTTCCTTCCTGTTGTCGTCCGGAGAGTATTCGCGTTGAGTGGAACAGCCGTATAAGAGTGGTTGGGCTAGCGGATTTAAAGGAATGTGGGTGCTGTGCCTTTAAGTTAGGACTCCTTGTGAAGGGATACGCAGAGCAGCGGCTGGATCGCAAGTTATTTCTAAAAATATATGGAAAGTAATGGGGGAGATATACGAAATGTATCTGCACGAAAACTCATATGCTCGCCAGATGTTTAGCTTGAGCAAAAACTTCAGATTCCTTAAAATTTCCGTTCTTATTATTTATTATTTCTGCTTTAGCGCAGGTTTTAATCAGAACTTTTTTCCCTTAATATAATTGGATGTGTGTATTTGCGCAGTGTTCGGCTCTCCCAGATTTTCTGGTTTTTAATTTTTTCCCTTTTCTTTTTTAATAAGAAAGTTTTGAAGTTTGATTACATTCTTTCCTATATCTTTCGATTCTATAAACTTTACATATTGGTCTAGACGCGTGGTTCCATCCAATTGAGAGTGAACTATTCGATTTCTTATATTAAGTTGCTCTTCCGGTGGCTCAGACACAGAAGTTGAGAAACGAGAAGTTTTAAAACACACACTATCCGGACAACTAGACATACTTTCGAAATCTGCTGTTTGCCGTTTTTTGCATAGGAAGGCTGTTTGTTTTTTGGTCAAATTGTAAAAATATTTGCTTTTCTTTAGGTGGGCTTTGGACTTCGCTTTTTCGTTGGCAATAACTATTTTTGTAAGACTTCTGGACATCTTTGTCCTGTAGTTATCGAAATTAATCGGGGTGCGCAACAAGATGTGATAACCTGCGTTCGTCTGAAAAATCTCAGATAGTTCGCCATCATTCAGCAACAAAATGTTTCTCTCAAAGACGAAAGACGTTTGTGTAAGGCTCAAGGGGNCCAAGTCACCGCCATGCCGGGCGGAGCAGCAGTCGGAGATCATTTTTGCCCATTCAGCAAACTCACATTTGCCTGATTGAATTAGATCCCTCGCATGCATTATCTTATTACCGCTTCCTGCCTCGTTCTCCTTACCACCCGTTCACGAAATGAACTACACCTGGTCCGAATTCGTGGGGTTTCACCAAGAAGTGGCTTGAAGGGAAGCGGGCATTTGTCATTGGCCCCCAGGGAAATCCCCAAAAAACGGGCCCCCCAGGATTCCATTTTCTTTTTCCGGGAGG

**> *pMos1-AeCpA-R2d2-3xP3-eCFP***

CCGGGTACCGAGCTCGAATTCGCCCTATAGTGAGTCGTATTACAATTCACTGGCCGTCGTTTTACAACGTCGTGACTGGGAAAACCCTGGCGTTACCCAACTTAATCGCCTTGCAGCACATCCCCCTTTCGCCAGCTGGCGTAATAGCGAAGAGGCCCGCACCGATCGCCCTTCCCAACAGTTGCGCAGCCTGAATGGCGAATGGACGCGCCCTGTAGCGGCGCATTAAGCGCGGCGGGTGTGGTGGTTACGCGCAGCGTGACCGCTACACTTGCCAGCGCCCTAGCGCCCGCTCCTTTCGCTTTCTTCCCTTCCTTTCTCGCCACGTTCGCCGGCTTTCCCCGTCAAGCTCTAAATCGGGGGCTCCCTTTAGGGTTCCGATTTAGTGCTTTACGGCACCTCGACCCCAAAAAACTTGATTAGGGTGATGGTTCACGTAGTGGGCCATCGCCCTGATAGACGGTTTTTCGCCCTTTGACGTTGGAGTCCACGTTCTTTAATAGTGGACTCTTGTTCCAAACTGGAACAACACTCAACCCTATCTCGGTCTATTCTTTTGATTTATAAGGGATTTTGCCGATTTCGGCCTATTGGTTAAAAAATGAGCTGATTTAACAAAAATTTAACGCGAATTTTAACAAAATATTAACGCTTACAATTTCCTGATGCGGTATTTTCTCCTTACGCATCTGTGCGGTATTTCACACCGCATATGGTGCACTCTCAGTACAATCTGCTCTGATGCCGCATAGTTAAGCCAGCCCCGACACCCGCCAACACCCGCTGACGCGCCCTGACGGGCTTGTCTGCTCCCGGCATCCGCTTACAGACAAGCTGTGACCGTCTCCGGGAGCTGCATGTGTCAGAGGTTTTCACCGTCATCACCGAAACGCGCGAGACGAAAGGGCCTCGTGATACGCCTATTTTTATAGGTTAATGTCATGATAATAATGGTTTCTTAGACGTCAGGTGGCACTTTTCGGGGAAATGTGCGCGGAACCCCTATTTGTTTATTTTTCTAAATACATTCAAATATGTATCCGCTCATGAGACAATAACCCTGATAAATGCTTCAATAATATTGAAAAAGGAAGAGTATGAGTATTCAACATTTCCGTGTCGCCCTTATTCCCTTTTTTGCGGCATTTTGCCTTCCTGTTTTTGCTCACCCAGAAACGCTGGTGAAAGTAAAAGATGCTGAAGATCAGTTGGGTGCACGAGTGGGTTACATCGAACTGGATCTCAACAGCGGTAAGATCCTTGAGAGTTTTCGCCCCGAAGAACGTTTTCCAATGATGAGCACTTTTAAAGTTCTGCTATGTGGCGCGGTATTATCCCGTATTGACGCCGGGCAAGAGCAACTCGGTCGCCGCATACACTATTCTCAGAATGACTTGGTTGAGTACTCACCAGTCACAGAAAAGCATCTTACGGATGGCATGACAGTAAGAGAATTATGCAGTGCTGCCATAACCATGAGTGATAACACTGCGGCCAACTTACTTCTGACAACGATCGGAGGACCGAAGGAGCTAACCGCTTTTTTGCACAACATGGGGGATCATGTAACTCGCCTTGATCGTTGGGAACCGGAGCTGAATGAAGCCATACCAAACGACGAGCGTGACACCACGATGCCTGTAGCAATGGCAACAACGTTGCGCAAACTATTAACTGGCGAACTACTTACTCTAGCTTCCCGGCAACAATTAATAGACTGGATGGAGGCGGATAAAGTTGCAGGACCACTTCTGCGCTCGGCCCTTCCGGCTGGCTGGTTTATTGCTGATAAATCTGGAGCCGGTGAGCGTGGGTCTCGCGGTATCATTGCAGCACTGGGGCCAGATGGTAAGCCCTCCCGTATCGTAGTTATCTACACGACGGGGAGTCAGGCAACTATGGATGAACGAAATAGACAGATCGCTGAGATAGGTGCCTCACTGATTAAGCATTGGTAACTGTCAGACCAAGTTTACTCATATATACTTTAGATTGATTTAAAACTTCATTTTTAATTTAAAAGGATCTAGGTGAAGATCCTTTTTGATAATCTCATGACCAAAATCCCTTAACGTGAGTTTTCGTTCCACTGAGCGTCAGACCCCGTAGAAAAGATCAAAGGATCTTCTTGAGATCCTTTTTTTCTGCGCGTAATCTGCTGCTTGCAAACAAAAAAACCACCGCTACCAGCGGTGGTTTGTTTGCCGGATCAAGAGCTACCAACTCTTTTTCCGAAGGTAACTGGCTTCAGCAGAGCGCAGATACCAAATACTGTTCTTCTAGTGTAGCCGTAGTTAGGCCACCACTTCAAGAACTCTGTAGCACCGCCTACATACCTCGCTCTGCTAATCCTGTTACCAGTGGCTGCTGCCAGTGGCGATAAGTCGTGTCTTACCGGGTTGGACTCAAGACGATAGTTACCGGATAAGGCGCAGCGGTCGGGCTGAACGGGGGGTTCGTGCACACAGCCCAGCTTGGAGCGAACGACCTACACCGAACTGAGATACCTACAGCGTGAGCTATGAGAAAGCGCCACGCTTCCCGAAGGGAGAAAGGCGGACAGGTATCCGGTAAGCGGCAGGGTCGGAACAGGAGAGCGCACGAGGGAGCTTCCAGGGGGAAACGCCTGGTATCTTTATAGTCCTGTCGGGTTTCGCCACCTCTGACTTGAGCGTCGATTTTTGTGATGCTCGTCAGGGGGGCGGAGCCTATGGAAAAACGCCAGCAACGCGGCCTTTTTACGGTTCCTGGCCTTTTGCTGGCCTTTTGCTCACATGTTCTTTCCTGCGTTATCCCCTGATTCTGTGGATAACCGTATTACCGCCTTTGAGTGAGCTGATACCGCTCGCCGCAGCCGAACGACCGAGCGCAGCGAGTCAGTGAGCGAGGAAGCGGAAGAGCGCCCAATACGCAAACCGCCTCTCCCCGCGCGTTGGCCGATTCATTAATGCAGCTGGCACGACAGGTTTCCCGACTGGAAAGCGGGCAGTGAGCGCAACGCAATTAATGTGAGTTAGCTCACTCATTAGGCACCCCAGGCTTTACACTTTATGCTTCCGGCTCGTATGTTGTGTGGAATTGTGAGCGGATAACAATTTCACACAGGAAACAGCTATGACCATGATTACGCCAAGCTCGAAATTAACCCTCACTAAAGGGAACAAAAGCTTTATGGTTTATGATTATCAATTTCGTTCCCATTGCTGGAATATCTTTCGGGTTGTTTGTTATGCCTCGCTTTTCACTGCGTGCTAGCAGTTCCCTTTTCCTTAGGAATGACCATGCGCACTTCCACTTTCGTGGGATAAAAATGGTAAATCCGGCTTTTTCTTAAAGCTACCAAATGATGTAGATAGGCATCCCACAGTACGGGCTCCAGTCGGTGAATATAGAAACTATCAGGTGTACAAGTATGAAATGTCGTTTTTTTTAAATCAAAAAACACGTTAAATTTTGTGGAAAAAGAAAAAATCATTTATTCAAAGTATTTGCCGTCGCTAGCTACACATTTTTCCCATCTCTCGGGCAATTTGTGGATTCCACGCCAGTAGAACTCATCGTCTTTTGCGGCGAACCATTCATCGAGCCATTTTTTCACACTTTCGTAAGAATCGAAGCGCTGCTCAGCGAGTGCGTGTCCCATCGAAGCGAATAGGTGGTAATCGGATGGGGCCAGGTCTGGTGAGTAAGCCGCATGCGGAAGCACTTCCCAATTGAGTGTTTCCAACGTGTCGCGAACCGCTCTTGCCGTATGTGATGGAGCGTTGTCATGGAGAAAAATGACCCTGTGTTGTCTTTTTTGATATTCCGGTCGTTTTCTCTGAAGCGCACGGTTCAAATTGATCAATTGTTGTTGGTAGCGTGCCGTATTCACCGTTTCGCCGGGTTTCAAGAGCTCATAGTAAATGACACCGCTCTGATCCCACCAAACACAGAGCATCGTCTTCTTGCCAAAGCGATTCGGTCGAGCAGTCGATGTGGCCGGTTGTCCAGGATCAACGTATGACTTTTTACGTTTAGGATTAACAAAAAAGATCCATTTTTCATCTCCAGTAACGATACGATGCAAAAACGACTTCCTTTTGTATCGTGAAAGCAAAATTTCGCATGTGTTTTTGCGCCTCTCCATCTGCCTCTCGTTCAACTCATGTGGCACCCATCTACCGACCTTCTGAATCTTTCCCATCTCTCGCAAGCGATTGGAAACTGCTTGTTGACTTACTTCCAACTGCTCTGCGAGTTGTTTTTGCGTTTGAGCATCGTCTTCATCCAATAATGCTTGCAGTTCGGCGTCTTCGTACCTTTTTGGCGGTTTTCCGTGCTCTTTGTCGTCGAAATTCGAGCTCGCCCGGGGATCTAATTCAATTAGAGACTAATTCAATTAGAGCTAATTCAATTAGGATCCAAGCTTATCGATTTCGAACCCTCGACCGCCGGAGTATAAATAGAGGCGCTTCGTCTACGGAGCGACAATTCAATTCAAACAAGCAAAGTGAACACGTCGCTAAGCGAAAGCTAAGCAAATAAACAAGCGCAGCTGAACAAGCTAAACAATCGGGGTACCGCTAGAGTCGACGGTACGATCCACCGGTCGCCACCATGGTGAGCAAGGGCGAGGAGCTGTTCACCGGGGTGGTGCCCATCCTGGTCGAGCTGGACGGCGACGTAAACGGCCACAAGTTCAGCGTGTCCGGCGAGGGCGAGGGCGATGCCACCTACGGCAAGCTGACCCTGAAGTTCATCTGCACCACCGGCAAGCTGCCCGTGCCCTGGCCCACCCTCGTGACCACCCTGACCTGGGGCGTGCAGTGCTTCAGCCGCTACCCCGACCACATGAAGCAGCACGACTTCTTCAAGTCCGCCATGCCCGAAGGCTACGTCCAGGAGCGCACCATCTTCTTCAAGGACGACGGCAACTACAAGACCCGCGCCGAGGTGAAGTTCGAGGGCGACACCCTGGTGAACCGCATCGAGCTGAAGGGCATCGACTTCAAGGAGGACGGCAACATCCTGGGGCACAAGCTGGAGTACAACTACATCAGCCACAACGTCTATATCACCGCCGACAAGCAGAAGAACGGCATCAAGGCCAACTTCAAGATCCGCCACAACATCGAGGACGGCAGCGTGCAGCTCGCCGACCACTACCAGCAGAACACCCCCATCGGCGACGGCCCCGTGCTGCTGCCCGACAACCACTACCTGAGCACCCAGTCCGCCCTGAGCAAAGACCCCAACGAGAAGCGCGATCACATGGTCCTGCTGGAGTTCGTGACCGCCGCCGGGATCACTCTCGGCATGGACGAGCTGTACAAGTAAAGCGGCCGCGACTCTAGATCATAATCAGCCATACCACATTTGTAGAGGTTTTACTTGCTTTAAAAAACCTCCCACACCTCCCCCTGAACCTGAAACATAAAATGAATGCAATTGTTGTTGTTAACTTGTTTATTGCAGCTTATAATGGTTACAAATAAAGCAATAGCATCACAAATTTCACAAATAAAGCATTTTTTTCACTGCATTCTAGTTGTGGTTTGTCCAAACTCATCAATGTATCTTAAAGCTTATCGATACGCGTACGGCGCGCCTAGGCCGGCCACCACTTTGTACAAGAAAGCTGGGTCGGCGCGCCCACCCTTGGTCGGCGCGCCCACCCTTGAGCTCGAATGGAACTGGCGGAACGGGACGGAATATTCGTGGAAGTGAATTTGAAGAAGTTTATTCTTCATAAGAACCAAAAATACAAATTGATAACTAAATCGTGTTTCTTTACGCCTGGATAAATGTAAAAATGTTTATTACGAAGATGTGTTGGCACCTCCAGCGTTGACAGAGTTTCGATTCCCAGCGAGGTTGTCTTCCCGTAGTGAAGACTGGCCATCCTTTCGCGTGATAGTAAACTATCTAGTAGGAATTACTTTTCTCCTTATTGAATACAATTAACCCATCTTTTATGCGTTTTGATTAATGTTCTTTTATTTGTTCTGTTGTAGCGATGGCTTTCGTTTGAATCCAAAATCCAGTACCGTTTTGAAGCCGAGATCGCATGCGCTCAGCTACCTATCTAGATCAATCAATGTAATCGAAATTAAGCATGTTCTTAAGATACTTGTAGACGCTCTCGTAAAACTTGTCTCCCTTTCCGAAAATGAAACAATCATATTCTGAATTCTGCAATTCGAATGTGAACAGTTTTCTTCCTCCGATCGTTACCAGATCGCTGTTGTCTCGTTTCATTTCGAATTTTAGTCCCAGAGCCTTGGGGATCAGGGTGCATTTGTCCTCCACGTTCAGCGTTTCGTCCAGAGCAATTCGTCGGGCGGCGGAAATTTTCGCTTCTTCCTGTTCTTCGAAGAACTGATGTCGGTCCGCAACCTTTACGCCCAGCTTTTTCTTGATGTCCGAGTTGATGAGTTCCCGGTAGGTCCGTATGATGTGCTCATCCTCGTTCAAATCGATCTCGGCGGCCAGATTGAGCGGCTGGACCTGCATTTCGCTGTCCTCCACTGGCATAGCTTGCAGCGTCTTAATCATCTCGTTGCACGCTGCCTGTTTGGCCCCTTTCTTCGTCTTGTGAATTCCCCGGCGGACAATCGCTCCGATGCGGCACTCATATTCAAACTCTGGAGCATGCGAAGGTCCCGAACTGCGGACACAGTTGAATTCGGGCATCCCGAAGTTCCGCTGCACGCAAATGTCCCGCACTTCGGTCACTTTATCCGAGGACAGGACCGCCAAAAAGTGCTCATCATTGTCCACGTCAATATCGCTCAGCCCTTTCTCGAACAGCAATTTCAACAACTTATAGGCAGCGTCATGTTTGGAATCCTTCTTTGACCGGCCGATTCCGCTGCTGGTGAATCCGAGGGCCAAAACCGAGGTGGTGAAAACTTTGGAATTCGGGTTTCCTCCGTCGATTTCCTCACCGGTAAATGTGTACAGCGGATGCGGCGCTTTTTGGGACACACACAGCTCTTGCAGCTCCGTTATTGGAGTCTTCGTGCTCAGGACTGGCTTTGAGGCCATggcgccTTTCCCAACTAACCGACTGCACACTAACCTGGCCAATTGCAATCAGGTGCCATTTATAGTTCGCTTGAGCACATCGTTAAGTGGAAATAGTTGTCTTATCAGCTACGGTACCTCATTGGAGCTTATACCGCGATTTAACTCCCGTTGGGTACTCGAAAGTAGAAGTCTGCCTTTGATCCATGCTGACGGATTCATTCTTAGTTTTATTTAACGTTTTTTCCACTGATTATGATAGGAATCGCTTTGACTTTTAAAATAAGCGATATTCAAAGCATGGTGGCCTTAAAGAACAGTAGGGTGAAGTCGGATAGGTTCACAGCAGGTATTCTAATTTAAACAATCTACCATGGAAAACAGGGCAGAATTTACAAATTCGGGACTCTGAGTCTACAGTTAGAAGGGCTTTTTTTAGTAATATTTGGTGATGTTGTAGCATTAGATAAAAGATAACTAAGGTGGCTAAGCATTAGATAAAAGAAACGTATCAACTTTCTGGTTTTGAATTGGGATATTAAAGTTCTCATTACAAAACGTAGTTTTAAACTTTTTTAAACTAGTTTTAAATGCTTCTTTGGTTCACTAGCTCAACAAGACTTTTAAAACTCAACCCATTACAATTCAAAAACAAATAAATGCTTAAACACTCTTAAAATATCTTGCTTTGAACGAATTTTTTTATTGACGAATTCCATGTCACGTTGGTCAGTCACAGATCTCTCCGTTGTGTAAAGTACAGTCACCCACAGTTATGGATCAACTAAGGGTCATTTTTCAGAACAAAACGTGATTAAAACTCATTAGTGGCGTTGTACAAAACAAAATCACCTCGCAGGCAGTGAAAAATATAGGGGAATTTACGTATTCTCTAAACAGCTGAACTTTTAAAAAGGCAGTTATACACAACAATAAATCACAACAATTAGCAGGAGACACCTGAACTACACTTGAGCAGTCTTCATTTATTGGTTATTATACACAAAACACTATTTTGTTCTCTAAATCAACTATTTTTCCTAAAGAAAGTCACGAGGCACTTGTTCTTTTATCGGCAATGCAATTACTATTGTCGGCAACTAAAATGTTCTCTTCGGCAATCCCTAACTGTGCAAAAACTAGTTTATGTATTGGTAACATTTCGTATTTGTTGACAAATCCCGAAATTAAACGTCGCTCATTATGTTCTACTTGTTGAAAGGGCCAATGCAGAAAAATACAGACTACACTGAGAACAACGGTCTTTCGGGAGAGGGAGGAGGTCGAAAATTGTACATTTTAGCTTTACGTCATAAATGGACGCTGCCTAATTTCGTTTTCATTTATTTCATCGAATATTTTTTGTATAAAATCTCACTATTGCAGCATTTTGGCTGTAAAAATTGCCATGTCATGGTTATAAACTTGCAGCAGGAGTGAATCCAACCAAGAACCAATCAACCAACCAAGAAGTTGCAGCGTGACATTTCTGTTGATTGATTCATCATATTGTGCTCTGCAAGAAAAATCCATGGAACGCGATTATCGGATTAATTGAATTTCAGAGTTGCGTTTGAATTGCGCGTTACTGCCGCCGCTGGATGTGAATTTCAAATGAGCACCGCAATATCATCCCTTGTACCCGCTCCTGCTCCACACCATTCAATTAACACGCATGACTTTTGTCGGGTGAAGGGGGCGGCCGCGGAGCCTGCTTTTTTGTACAAACTTGTGGCCGGCCGATCTCGTCGACGTCAAAATCACCACTTTTGAAGCGTTGAAACCACCGTTCACACGTTTTCACAGTTGGTACTTGTTCGCCAAAGGCTTCAACAAGCATTCGGTGCGATTCCGCAGCTGTTTTCTTCAAATGAAAACAGAAAATTAATACTGTCCGCGTTTGCTCTTTATTCGGCACGAAACTCGACATGTTGACTGCACTGAGAGTAAACAATTATGACGCTCAATTCGCGCCAAACTATGGTGGTTCGACAGTCAAGGTTGACACTTCACAAGGTCAAAGTTTTATGACAATCGATAAATATTTACGTTTGCGAGACATCTATATGTTCGAACCGACATTCCCTACTTGTACACCTGGTAAAAAATTAAAACTAGGTCCCCGTTCTTACGCATATATACACAGGTACGTGGGTGTTAATTAATTACTTTAAAACATTAAGGAACTTCGATTGGCATTTAACAAGGAAACTAACATCATATTGTTATACAAATAAAAGTTGCTCATTTCTTACTCGTAATTCGAAACCTTTCTCGGTTCTTTTTTTTACCATACTATCACTTTAAAGTGGTTTTAACCAACATGCATACATTTCGTTTTTTTTTCGTTTTACTCGTCTGCGTTCGCTCTTTGTTTTTTAACCAAAGTAAATCTGACTCAGGGAAGTCACTCAAAACATGTTTGCCTGAGCTCTCGTATTCCAAATGGAACTCCGGGAAAAATATATGCTCAAAAGTGGCTCAAAACTGTATCTATTCGTATTTTGATATACACAATAATAAAACTGCTCGAAGAACTCCAATCTGTTACACAATGCCTGGTCATTGTTTACATTTAGCTCACTTGTGTACTTATGTGCACCCATACACACCCATGTGTAGATAAAGGATGGCTTATCTTATCACTGACAAAACAGCGGAGCATGCAAGAATGCTGTTTGTCTTTGTCCATTGGCTTCGATTAAGTAGTCGAAAAGGAAAACGGTAAATGGAATGCATAACTTTGCCCTCTCACTCCCTCTAATCGGACGGAAGAGTCGGACGTTATGACCTGTCCTGATTTGGAGAAATCTTTTCTATATTTCATTTATTTCGCTTACTTCCCTACTTCTTTCAAATGCACTAATGCGAAAAACATTATTTTTTTCAAATTAAATGGTCAAGTGTGCTTCTAATGAAATCCATAGTCTCAATACATACATATATGCAGTCGGCAAAAAAGGAGGCACATACTCGTAGAAATTTTTCATTGCTCTGTATATGGATTTGTAAATGGTATAAAAAGCATATATCAGTATCAGTTCTCTTCCCATTCCCGCTCTCAGTGCGCTCTCTCGGTTGAGCAGTTTTCGAGCCTCTCGAGTTCGAGTTCACCGAGTTCGAGTTGCGACGGTTCGCCCACTCAGTTTGCCCACAGCTCTGAATGCGAGCGTAACGATCGCAAGCCGCCTGTCTTGCAGTGAAGTGCATTTTCTCAAGCCGCTTCCTCGATTTGTATTTGGTTGGACAACAGCCCATAACTTGAACCCGTCTTTAATGCCCGTATCTCTGCGAGAGTGAGTTTCCAATGGCGATTTGCCGAGTGCGCGTGTGCAGGGCACCCTTTCCTTCCTGTTGTCGTCCGGAGAGTATTCGCGTTGAGTGGAACAGCCGTATAAGAGTGGTTGGGCTAGCGGATTTAAAGGAATGTGGGTGCTGTGCCTTTAAGTTAGGACTCCTTGTGAAGGGATACGCAGAGCAGCGGCTGGATCGCAAGTTATTTCTAAAAATATATGGAAAGTAATGGGGGAGATATACGAAATGTATCTGCACGAAAACTCATATGCTCGCCAGATGTTTAGCTTGAGCAAAAACTTCAGATTCCTTAAAATTTCCGTTCTTATTATTTATTATTTCTGCTTTAGCGCAGGTTTTAATCAGAACTTTTTTCCCTTAATATAATTGGATGTGTGTATTTGCGCAGTGTTCGGCTCTCCCAGATTTTCTGTTTTTAATTTTTTCCCTTTTCTTTTTTAATAAGAAAGTTTTGAAGTTTGATTACATTCTTTCCTATATCTTTCGATTCTATAAACTTTACATATTGGTCTAGACGCGTGGTTCCATCCAATTGAGAGTGAACTATTCGATTTCTTATATTAAGTTGCTCTTCCGGTGGCTCAGACACAGAAGTTGAGAAACGAGAAGTTTTAAAACACACACTATCCGGACAACTAGACATACTTTCGAAATCTGCTGTTTGCCGTTTTTTGCATAGGAAGGCTGTTTGTTTTTTGGTCAAATTGTAAAAATATTTGCTTTTCTTTAGGTGGGCTTTGGACTTCGCTTTTTCGTTGGCAATAACTATTTTTGTAAGACTTCTGGACATCTTTGTCCTGTAGTTATCGAAATTAATCGGGGTGCGCAACAAGATGTGATAACCTGCGTTCGTCTGAAAAATCTCAGATAGTTCGCCATCATTCAGCAACAAAATGTTTCTCTCAAAGACGAAAGACGTTTGTGTAAGGCTCAAGGGGCCCAAGTCACCGCCATGCCGGGCGGAGCAGCAGTCGGAGATCATTTTTGCCAATTCAGCAAACTCACATTTGCCTGATTGAATTAGATCCCTCGCATGCATTATCTTATTTAGCGCTTCCTGCCTCGTTCTCCTTACCACCCGTTCACGATATGAACTACACCTGTCCGATTCGTTGTGCTTCACCAGGATGTGTCTGCAGCGGAGCGTGCATTTGTCATTGCACTCAGGGTAGTCCCCAAGAACGGCGCCCCAGGCATTCCTATTCTTTTCTCGGTAGGGGCTTGGAGGCAGTGTGAAATATACCTTCCTGGTAATTGTGTCGTAGAAGTAGCACTCCTTGGTACTGTGAACAATTCGCTCTTCCCATCCAAGGGGCAGCTTGTTAGGCCTCTCAGAATTATGACGTTCGCCAGTTAGGATCGAGAGCCCACTGTCCGCATCACCATAGGAGTCGCTTTTAACGTTATCACTCGAATATTTACTTTTAGAGCTCGCGTCTAACCAACCCCAAGAGTGGTCACAAGTTTGCTGATCTTCATTTTGGCCATCTTCTATTTTAAATATATCAAACTTGCATAGCGCCGGCTTCATGGTGTTCATGTTTGCACAAACCAGACGGTATAGGTATTTATGTGTTAAGTATAGATACTGAATAGGTTACTGCTTTGAGCCAAACTCTTAAAGAAAAATAAAGCAATGCAATCTTGTTTGTTTTACAGCTCGGCTTTAAGGCATATATTTTTAATTATGTTTTATTTTCATGCTTTTATTTTTTTTTAGGTTTCCGAAATAGTTATGAATCTTATACTGACTGTAACCACGGCTCTGCACCTAGTTTTGGTGGTATTGGCGCACAATCCGCCGTCGATTCGTGGCGAAAGCCATTCTGATGGCATTCGTCTGGTCATGGAAAAGAGGTCGGGCGATGTAGTACACATTCGTCCAGCTCGGGGCACCCTGGAGGACGAAACAGTCGCATCCACATCTACAACAAAAAGTGACACCCACCGCACCAGGAATAATCGTAAGCCGAACAAGCCGCAAGAGCACTCTCAGCAGGTTGGGGCCCACAAGTCGGGATCTAGAAAACTAGTGGTTGCGGAAAATGGCGGGGCACTGTCATCACAGCTACCACAACCAAATCACAAACAAGGCCACAAGAACACTGATAAGCAACAACGAGGAGGAGGATCC
