# Supplementary figures and images for: The Aedes aegypti siRNA pathway mediates broad-spectrum defense against human pathogenic viruses and modulates antibacterial and antifungal defenses
Source: PLoS Biol. 2022 Jun 9;20(6):e3001668. doi: 10.1371/journal.pbio.3001668 (PMC9182253; doi:10.1371/journal.pbio.3001668)

Fig. 1D

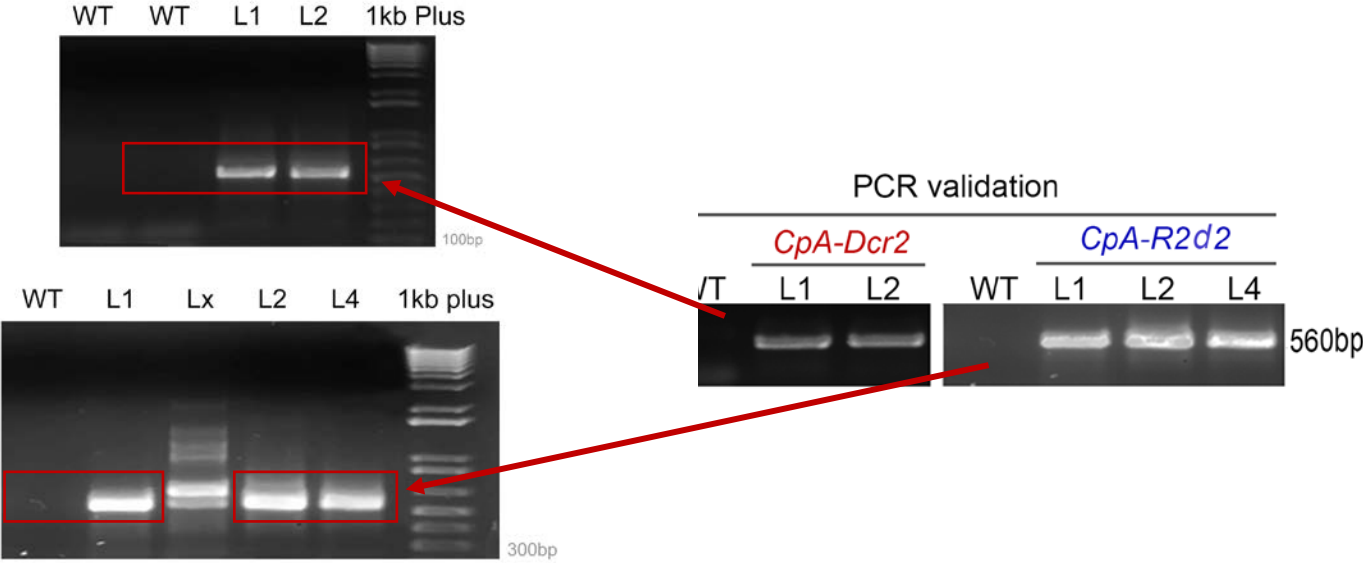

Lx: unconfirmed line

Supplement: S1 Raw Images — (PDF) [file pbio.3001668.s012.pdf]
